# Controlling Triboelectric Charge of MOFs by Leveraging Ligands Chemistry

**DOI:** 10.1002/advs.202404993

**Published:** 2024-07-12

**Authors:** Muhammad Noman, Qazi Muhammad Saqib, Shahid Ameen, Swapnil R. Patil, Chandrashekhar S. Patil, Jungmin Kim, Youngbin Ko, BongSoo Kim, Jinho Bae

**Affiliations:** ^1^ Department of Ocean System Engineering Jeju National University Jeju 63243 Republic of Korea; ^2^ Department of Chemistry Ulsan National Institute of Science and Technology (UNIST) Ulsan 44919 Republic of Korea

**Keywords:** materials chemistry, metal–organic frameworks, organic ligands, triboelectric charge, triboelectric nanogenerator

## Abstract

Metal–organic frameworks (MOFs) have emerged as promising materials for triboelectric nanogenerators (TENGs), but the effects of ligand choice on triboelectric charge remain underexplored. Hence, this paper demonstrates the effect of single, binary, and ternary ligands on TENG performance of cobalt/cerium‐based (Co─Ce) bimetallic MOFs utilizing 2‐methylimidazole (2Melm), terephthalic acid (BDC), and benzene tricarboxylic acid (BTC) as ligands. The detailed structural characterization revealed that varying ligand chemistries led to distinct MOF features affecting TENG performance. Single ligand bimetallic MOFs (designated as CoCe‐2MeIm, CoCe‐BDC, CoCe‐BTC) has lower performance than binary ligand (designated as CoCe‐2MeIm‐BDC, CoCe‐2MeIm‐BTC, CoCe‐BDC‐BTC) and ternary ligand MOFs (designated as CoCe‐2MeIm‐BDC‐BTC). Among all, the binary ligand MOF, CoCe‐2MeIm‐BTC, shows the best results (598 V, 26.7 µA) due to the combined effect of imidazole ring and (─COO─) groups. This is attributed to lone pairs on nitrogen atoms and a delocalized π‐electron system in imidazole system in this material. CoCe‐BTC has the lowest results (31 V, 3.2 µA) due to the bulkier nature of the electron‐withdrawing (─COO─) groups and their impact on the π‐electron system of the benzene ring. This study showcases the potential of ligand chemistry manipulation to control triboelectric charge and thereby enhance MOF‐based TENG performance.

## Graphical abstract

To enhance metal–organic framework (MOF)‐based triboelectric nanogenerator (TENG) performance, it explored the effects of single, binary, and ternary ligands for triboelectric charge. Especially, cobalt/cerium‐based (Co─Ce) bimetallic MOFs utilizing 2‐methylimidazole (2Melm), terephthalic acid (BDC), and benzene tricarboxylic acid (BTC) ligands are considered, and the binary ligand MOF (CoCe‐2MeIm‐BTC) shows the best results (598 V, 26.7 µA) due to the combined effect of imidazole ring and (─COO─) groups. Hence, these results can boost TENG performance while keeping stable temperature and humidity characteristics.

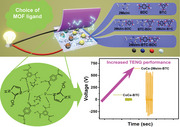

## 1 Introduction

In order to address current issues with energy generation and sustainability, research on energy‐harvesting gadgets and devices is crucial.^[^ ^1^, ^2^, ^3^ ^]^ Triboelectric nanogenerators (TENG) can help overcome these challenges by providing decentralized and sustainable power sources for self‐powered wearable electronics and Internet of Things (IoT) devices.^[^ ^4^, ^5^, ^6^ ^]^ The TENG uses triboelectrification and electrostatic induction to convert mechanical energy into electrical power.^[^ ^7^ ^]^ However, a recent focus has been on increasing the power density and output voltage of TENGs. TENGs' electrical output performance depends on a number of factors, including materials, device design, and operating conditions.^[^ ^8^, ^9^ ^]^ In TENGs, energy is generated due to the relative motion of two different materials when they are brought into contact. This phenomenon has been well‐documented in the triboelectric series. Furthermore, the materials are stacked in a triboelectric series according to their electron affinities. The greater the difference between two materials in electron affinity, the more substantial output performance can be obtained. Exploring materials with varying properties and functionalities is essential to commercializing the TENG devices. It is also noteworthy that such materials can be positioned at the extremes of the triboelectric series so that they can be employed as tribopositive and tribonegative layers. The triboelectric series primarily focuses on polymers and a few other materials; however, extending it to include a wider range of materials, particularly (metal organic frameworks) MOFs, would add to its significance. Compared to polymeric materials, MOFs have a number of benefits, including heterogeneity, multifaceted structures, and ease of functionalization.^[^ ^10^, ^11^ ^]^ Moreover, certain functional groups that are frequently present in the organic ligands of MOFs facilitate the grafting of MOF structures either during or after synthesis. Furthermore, using multiple ligands or metal ions can result in complex, MOF‐on‐MOF structures.^[^ ^12^, ^13^ ^]^ These structures consist of one MOF grown on the surface of another pre‐existing MOF, creating a composite of more than one MOF framework.^[^ ^14^ ^]^ This can significantly alter MOFs' physicochemical and triboelectric charge properties by introducing chemically stable polar and nonpolar functionalities.^[^ ^15^ ^]^ MOFs within these categories show significant promise in enhancing TENG performance because of their humidity and temperature stability.^[^ ^16^ ^]^

MOF structures are formed through coordination bonds between metal ions and organic ligands.^[^ ^17^ ^]^ MOFs possess high specific surface area, tuneable porous structures, variable morphologies, and facile movement of surface electrons and ions. These characteristics contribute to their triboelectrification and electrostatic induction properties. Modifying the shape, aggregation, composition, and dimensions of metal ions, solvents, and organic ligands can yield diverse MOF structures. Bimetallic MOFs exhibit diverse architectures driven by controlled compositions and structures. These MOFs demonstrate synergistic effects and enhanced properties, compared to their monometallic counterparts, for various applications including energy storage and conversion.^[^ ^18^, ^19^ ^]^ Likewise, employing single, binary, and ternary ligands and their interactions with solvents and various metal ions can cause different reaction chemistries and varying properties in MOFs. An important aspect that has been relatively overlooked in this regard is the significant impact of metallic and organic/ligand counterparts on the triboelectric performance of MOFs within TENGs.^[^ ^20^, ^21^, ^22^ ^]^ To provide context, it is worth noting that nearly 90000 different MOF structures have been synthesized to date. Among these, three ligands have emerged as exceptionally versatile and commonly used: 2‐methylimidazole (2Melm), terephthalic acid (BDC), and benzene tricarboxylic acid (BTC).^[^ ^23^ ^]^ 2‐MeIm is of particular importance due to its specific structural features such as highly delocalized π‐electron system, and its capacity to both donate and accept electrons. Therefore, understanding the influence of these ligands in MOFs for enhancing TENG performance is crucial.^[^ ^24^, ^25^ ^]^

Additionally, by examining the structural and chemical characteristics of MOFs, a mechanism that influences the generation and manipulation of triboelectric charge due to ligand chemistry can be clarified, ultimately resulting in improved TENG performance.^[^ ^26^ ^]^ Therefore, the study of various physiochemical and structural aspects of MOFs inherits great potential to extend the triboelectric series, allowing for exploring a broader range of materials for improved TENG performances.^[^ ^27^ ^]^

In this study, we explored how different ligands in bimetallic cobalt/cerium (Co/Ce) MOFs impact TENG performance. We focused on three widely used ligands viz. 2Melm, BDC, and BTC. The structural analysis revealed that these ligands lead to MOFs with distinctive structures and functionalities. The structural features of these MOFs, when utilized as tribopositive materials in TENG fabrication, significantly influenced the performance of the devices. Specifically, the bimetallic Co/Ce MOF containing both 2‐MeIM and BTC ligands, i.e., CoCe‐2Melm‐BTC outperformed others, generating 598 V and 26.7 µA, while the one based on BTC only, i.e., CoCe‐BTC yielded the lowest results at 31 V and 3.2 µA. Device performance of CoCe‐2Melm‐BTC was enhanced by the combined action of the electron withdrawing (─COO─) groups from BTC and the electron‐rich attributes of the imidazole ring in 2‐MeIm. The electronegativity of nitrogen, a delocalized π‐electron system, and lone pairs on nitrogen atoms all contribute to imidazole‐containing MOFs' high electron‐donating capabilities. On the other hand, poor triboelectric performance of the device based on CoCe‐BTC was caused by the electron‐withdrawing effect of the (─COO─) groups and bulkier nature of BTC. The impact of ligand chemistry in MOFs on controlling the triboelectric charge for improved TENG performance is highlighted in this work.

## 2 Results and Discussions

**Figure 1:**
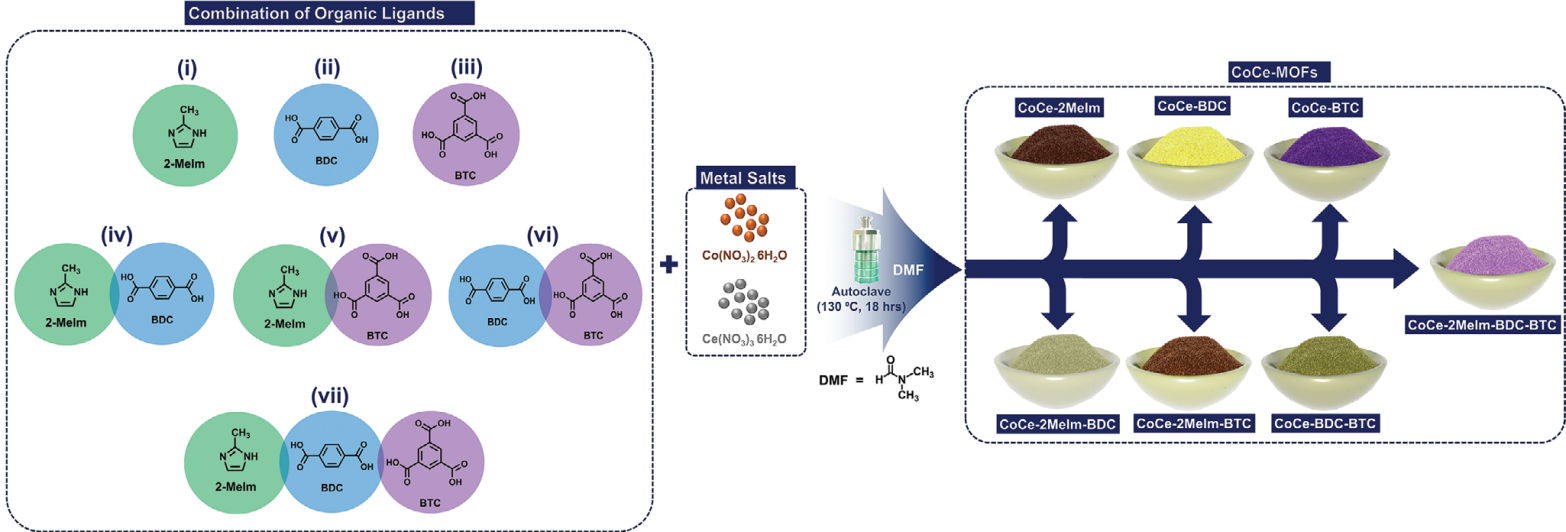
Solvothermal synthesis of CoCe‐MOFs by utilizing various combinations of 2Melm, BDC, and BTC organic ligands.

CoCe‐MOFs were synthesized using unary, binary, and ternary ligands via a facile solvothermal approach to study the impact of ligand chemistry on the triboelectric performance of the TENG as shown in **Figure** **1**.

**Figure 2:**
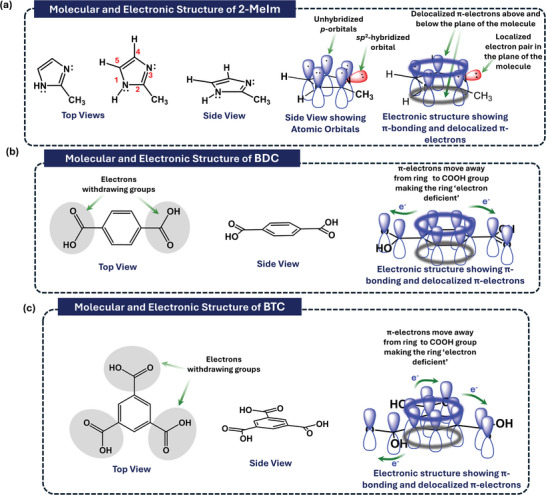
Schematic diagram illustrating the molecular and electronic structures of 2Melm, BDC and BTC suggesting the role of lone pairs for charge donation in TENGs.

The complete synthesis procedure for seven distinct MOF samples is detailed in the experimental section. The ligands used for the synthesis of the MOFs have specific structural and electronic features. The imidazole ring in 2Melm contains two nitrogen atoms with lone pairs of electrons one of which is readily available for donation to other atoms or molecules while the other is involved in the electronic resonance of the delocalized electrons as shown in **Figures** **2**a and S1a (Supporting Information).^[^ ^28^ ^]^ Moreover, the electronic structure of imidazole involves a delocalized π‐electron system. This π‐system is resonance stabilized with high electron density within the ring. The electron density of one of the two nitrogen atoms of the ring is out of the ring and could be donated to easily to an electron acceptor such as a metal ion^[^ ^29^, ^30^ ^]^ On the other hand, BDC and BTC contain electron withdrawing carboxylic acid groups that decrease electron density on the benzene rings of these ligands decreasing the availability of transferable charge carriers as shown in Figures 2b,c and S1b,c (Supporting Information), respectively.

**Figure 3:**
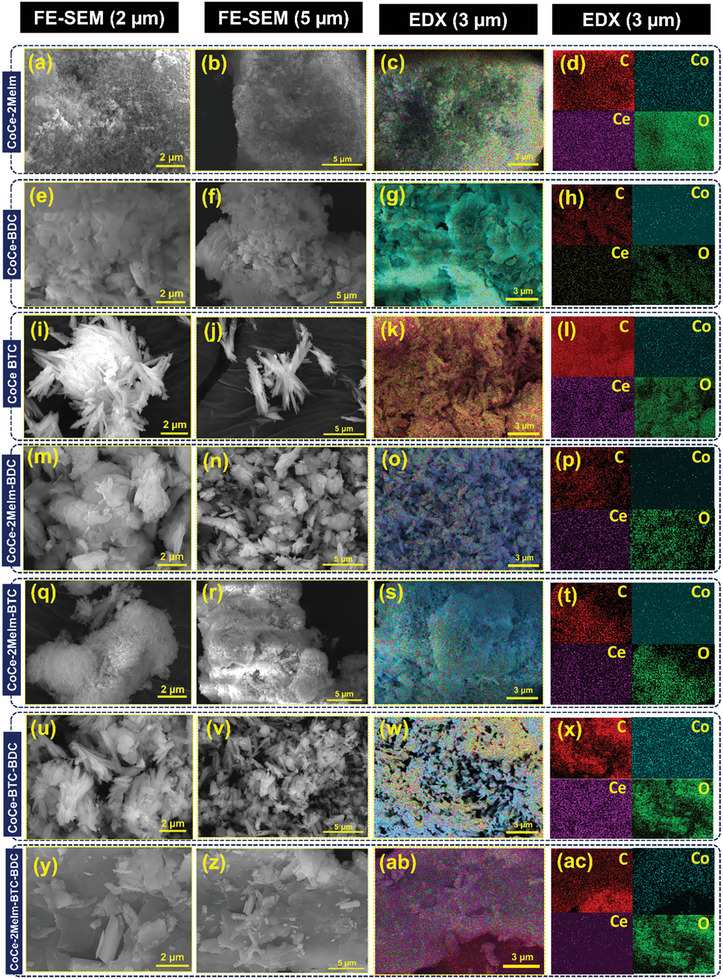
FE‐SEM images of CoCe‐MOFs at 2 and 5 µm magnification and corresponding EDX mappings at 3 µm; a–d) CoCe‐2Melm, e–h) CoCe‐BDC, i–l) CoCe‐BTC, m–p) CoCe‐2Melm‐BDC, q–t) CoCe‐BTC‐BDC u–x) CoCe‐2Melm‐BTC, and (y‐ac) CoCe‐2Melm‐BTC‐BDC MOFs.

Once part of the MOFs these ligands impart specific structural, charge generation and distribution properties to the materials. The synthesized CoCe‐MOFs were employed, in powder form, as tribopositive materials in the TENG, while PTFE was used as the tribonegative layer. Morphological features of MOF powders can affect the TENG performance for various reasons, such as surface area contact, interfacial contact, charge transfer conduction/resistance, and particle alignment and distribution. To investigate this, **Figure** **3** displays the FE‐SEM images along with corresponding EDX mappings of all samples. Notably, each sample exhibits distinct surface morphologies, implying the influence of successful coordination of Co^2+^ and Ce^3+^ with different ligand combinations. The CoCe‐2Melm sample manifests granular‐shaped particles (Figure 3a,b), CoCe‐BDC showcases a petal‐like morphology (Figure 3e,f), and CoCe‐BTC presents agglomerated straw sheaves‐like morphology (Figure 3i,j). In the case of CoCe‐2Melm‐BDC, it demonstrated agglomerated flakes‐like morphology in which small‐sized detached flakes were distributed among larger flakes (Figure 3m,n). In CoCe‐2Melm‐BTC, nano‐sized round particles were distributed between micro‐rods, contributing to a loosely packed morphology as shown in Figure 3q,r. For CoCe‐BTC‐BDC, very thin nanorods were formed, which had penetrated each other, resulting in a tightly packed morphology as shown in Figure 3u,v. CoCe‐2Melm‐BTC‐BDC structure exhibited a morphology of smooth flakes interspersed with smaller flakes and nanorods distributed across their surface as shown in Figure 3y,z. This intricate arrangement suggests that the smaller flakes and nanorods possibly originated from the growth processes occurring on the larger flakes. To confirm the elemental composition of all MOF samples, EDX was performed as shown in Figure 3 and Figure S4 (Supporting Information). All the samples showed the presence of Co, Ce, O, and C moieties. In the case of CoCe‐2Melm‐BTC, during growth, the micro‐rods intersect with nanoparticles, which can help impede the growth of these micro‐rods. This, in turn, reduces the longitudinal dimensions of the micro‐rods, leading to a nanometer length scale, which is more beneficial in maintaining a higher contact area. This arrangement proves advantageous during contact separation, enhancing electrostatic induction and triboelectrification, benefiting the favorable triboelectric charge distribution. On the other hand, in the case of BTC alone, agglomerated straw sheaves‐like morphology possesses a smaller contact area compared to granular‐shaped and petal‐like morphologies, indicating limited surface charge distribution, contributing to a drop in TENG output performance.^[^ ^31^ ^]^ Moreover, straw sheaves may exhibit a more uniform or smoother surface, potentially minimizing the triboelectric charge generation efficiency compared to irregular or rougher surfaces found in granular or flower petal‐like morphologies.

**Figure 4:**
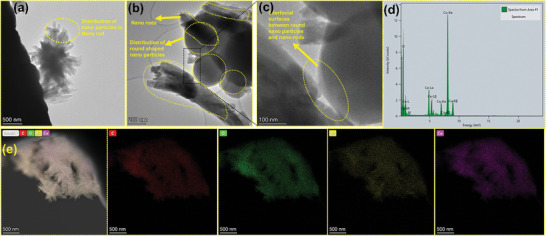
For CoCe‐2Melm‐BTC: TEM images at a) 500 nm, b) 200 nm, c) 100 nm, d) EDX analysis, and e) elemental mapping of C, O, Co, and Ce elements.

Further confirmation of round‐shaped nanoparticle distribution within the nanorods of CoCe‐2Melm‐BTC is obtained through HR‐TEM imaging, as shown in **Figure** **4**a–c. The images reveal fine nanoparticles distributed within the rods, with some rods measuring within the 100–120 nm range. Increased interfacial resistance between spherical particles and longitudinally oriented nanorods may enhance TENG performance by promoting greater charge separation and improved electron transport. Additionally, these nanorods possess sharply defined tips and edges which are beneficial for concentrated and interfacial triboelectric charge transfer, contributing favorably to high triboelectric performance. Furthermore, Figure 4d,e shows the high resolution EDX analysis with corresponding elemental mapping of CoCe‐2Melm‐BTC, confirming the presence of constitutional elements such as C, O, Co, and Ce in the MOF structure.

**Figure 5:**
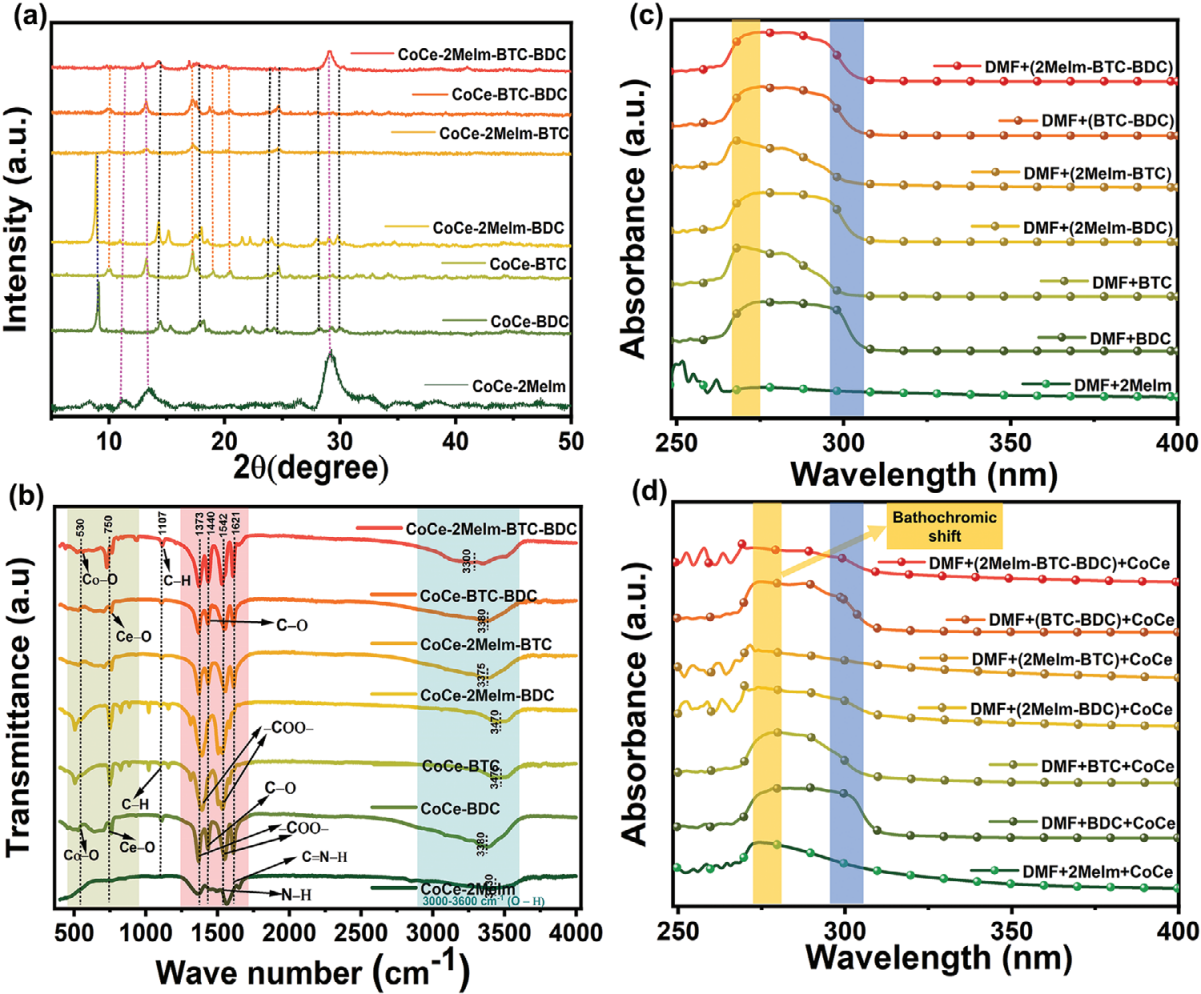
a) XRD spectrums and b) FT‐IR spectrums of all MOF samples, UV–vis spectra of CoCe‐2Melm, CoCe‐BDC, CoCe‐BTC, CoCe‐2Melm‐BDC (1:1), CoCe‐2Melm‐BTC (1:1), CoCe‐BTC‐BDC (1:1), and CoCe‐2Melm‐BTC‐BDC (1:1:1) ligands in (c) DMF and d) DMF with Co and Ce ions.

**Figure** **5**a demonstrates the room‐temperature powder XRD patterns of the seven CoCe‐MOFs and MOF‐on‐MOF structures. The XRD patterns illustrate high‐pitched Bragg reflections, signifying the crystalline nature of the MOF samples. Furthermore, upon conducting a comparative analysis of all the MOFs, the XRD patterns revealed varying peak intensities, with some similar peaks detected across all the MOF specimens. The PXRD patterns of bimetallic MOFs such as CoCe‐2Melm, CoCe‐BDC, and CoCe‐BTC were compared with those reported in the literature. There was a slight shift in the peaks due to the formation of bimetallic MOF and MOF on MOF structures. For example, the PXRD pattern of CoCe‐2Melm exhibit characteristic peaks at 10.5°, 12.9°, 15.8°, 25.3°, 28.5°, 32.1°, 34.8°, and 37.8° confirming the formation of CoCe‐2Melm bi‐metallic MOF.^[^ ^32^, ^33^, ^34^ ^]^ Moreover, the PXRD pattern of CoCe‐BDC demonstrated characteristic peaks at 9.2°, 11.9°, 14.4°, 15.7°, 18.2°, 18.9°, 23.8°, 24.4°, 28.3°, 29.4°, and 30.3° confirming the successful formation of bimetallic CoCe‐BDC MOF.^[^ ^35^, ^36^, ^37^, ^38^, ^39^ ^]^ Similarly, the PXRD pattern of CoCe‐BTC showed characteristic peaks at 10.2°, 13.2°, 17.1°, 17.9°, 20.5°, 24.3°, 25°, and 34.3° confirming the formation of bimetallic CoCe‐BTC MOF.^[^ ^40^, ^41^, ^42^ ^]^ For the peak identification of MOF‐on‐MOF structures such as CoCe‐2Melm‐BDC, CoCe‐2Melm‐BTC, CoCe‐BDC‐BTC, and CoCe‐2Melm‐BDC‐BTC, a stacking comparison of the XRD graphs of single‐ligand bimetallic MOFs was carried out. Additionally, simulated pattern patterns of 2Melm (CCDC No. 2095301), BDC (CCDC No. 638866) and BTC (CCDC No. 1274034), ligands have been included in the PXRD graphs shown in Figures S5–S8 (Supporting Information). This analysis revealed that for CoCe‐2Melm‐BDC, some peaks originate from CoCe‐2Melm and others from CoCe‐BDC, as shown in Figure S5 (Supporting Information). Similarly, for 2Melm‐BTC, some peaks originated from CoCe‐2Melm and others from CoCe‐BTC, as presented in Figure S6 (Supporting Information). Likewise, for CoCe‐BDC‐BTC, the XRD peaks came from CoCe‐BDC and CoCe‐BTC, as shown in Figure S7 (Supporting Information). Also, for CoCe‐2Melm‐BDC‐BTC, the PXRD pattern confirmed the presence of characteristic peaks of CoCe‐2Melm, CoCe‐BDC and CoCe‐BTC bimetallic MOFs as shown in Figure S8 (Supporting Information). Consequently, the PXRD analysis confirmed the successful formation of bimetallic and MOF‐on‐MOF structures. Moreover, when ligands combine, certain particles experience enhanced growth, while others impede the growth of adjacent particles. This synergistic effect of ligands leads to diverse particle sizes and lattice parameters in complex MOF‐on‐MOF structures.^[^ ^43^, ^44^ ^]^

Figure 5b shows the FT‐IR results for the seven MOFs. Normally, the bands correspond to the M─O bond are present in 400–600 cm^−1^ range. Two peaks ≈530 and 750 cm^−1^ were found corresponding to the stretching vibration of the Co─O and Ce─O bonds, respectively.^[^ ^37^, ^45^ ^]^ For the presence of BTC in CoCe‐BTC, CoCe‐2MeIm‐BTC, CoCe‐BTC‐BDC, and CoCe‐2Melm‐BDC‐BTC, strong transmittance bands in the range of 1340–1440 cm^−1^ and 1480–1580 cm^−1^are obtained. These bands are assigned to the asymmetric and symmetric stretching vibrations of carboxylate groups (─COO─) due to the presence of BTC linkers.^[^ ^46^, ^47^ ^]^ The interaction of these functional groups with the surrounding environment can alter the surface chemistry of MOFs, affecting the triboelectric charge collection performance. The band ≈1107 cm^−1^ might be associated with C─H in‐plane bending vibration,^[^ ^46^ ^]^ whereas broad asymmetric bands ≈3350 cm^−1^ correspond to the stretching vibrations of O─H bonds of the M(OH)_2_ moieties in all MOFs.^[^ ^48^ ^]^ In the case of BDC, asymmetric and symmetric stretching vibrations of carboxylate groups (─COO─) are represented by similar bands as of BTC, except for introducing a sharp band at ≈1430 cm^−1^. These bands can be assigned to the presence of C─O groups and confirm the deprotonation of BDC linkers.^[^ ^49^, ^50^ ^]^ In the FTIR spectra of the 2‐MeIm‐based MOFs, the bands at 1560 and 1630 cm^−1^ resulted from the bending and stretching of C═N─H and other bonds of the imidazole ring, respectively.^[^ ^51^ ^]^ The characteristic peak of the N─H stretching in 2Malm at 1544 cm^−1^ indicates the formation of the N─M bond.^[^ ^52^ ^]^ Hence, FT‐IR spectra prove the presence of electron‐donating groups in MOF structures responsible for triboelectric performance.

After that, UV–vis spectroscopy was employed to comprehend the intermolecular interaction mechanism among Co^2+^ ions, Ce^3+^ ions, unary, binary, and ternary ligands and with the solvent molecules, as shown in Figure 5c,d. The UV–vis spectroscopy was conducted before the solvothermal reaction, involving fourteen solutions prepared in DMF solvent. Consequently, seven solutions were prepared with BTC, BDC, and 2Melm ligands dissolved separately and in binary and ternary combinations in DMF. In comparison, the remaining seven solutions had Co and Ce ions dissolved alongside these ligands. These solutions exhibited different colors due to the various combinations of ligands and ionic salts, as illustrated in Figure S3 (Supporting Information). The resultant graphs of UV–vis absorption spectra before and after adding Co^2+^ and Ce^3+^ ions are shown in Figure 5c,d. Upon adding Co^2+^ and Ce^3+^ ions to the ligand solutions, a bathochromic shift was observed in the absorption peak of ligand molecules, as shown in Figure 5c. This redshift stemmed from the decreased interaction between ligands and solvent molecules,^[^ ^53^ ^]^ with the interaction between metal ions and ligands (M‐L) becoming dominant. In this interaction, the d orbitals of central metal ions, Co^2+^ and Ce^2+^, usually split up. As a result of this d–d transition, the light absorption efficiencies of MOF change.^[^ ^25^ ^]^ Within a specific molecular interaction, metal ions break the DMF carbonyl (C═O) bonds and form coordination bonds with the free atoms of the ligands. In the cases of BTC and BDC, the metal ions primarily form bonds with the carboxylate (─COO─) groups, whereas, in the case of 2Melm, bonding occurs primarily through nitrogen atoms.^[^ ^54^, ^55^ ^]^ As a result of this bonding between ligands and metal ions, the molecular structure grows three‐dimensionally, forming a framework structure.^[^ ^56^ ^]^ When different ligands are used in MOFs, it alters their electronic structure, leading to changes in their orbital compositions. Consequently, this affects the electron donation and acceptance energies of their metal centers, such as HUCO (Hexa‐µ2‐chloride) and LUCO (Lanthanide Coordination Sphere).^[^ ^57^ ^]^

As a result, it may impact the triboelectric charge generation and collection due to modifications in the electronic properties of the MOFs.^[^ ^58^, ^59^ ^]^ To substantiate this further, when Co^2+^ and Ce^2+^ ions were added to unary, binary, and ternary ligands, a clear change in the absorption edge was observed, especially more pronounced in the binary and ternary ligands. This change could be associated with complex structural formation and abundance of carbonyl (C═O) bonds linked with metallic ions.^[^ ^60^ ^]^ Specifically, in the case of BTC only, three electron‐withdrawing (─COO─) groups are attached to the benzene ring.^[^ ^61^ ^]^ The ligands' bulky and stable nature also contributes to lower electron donation efficiency. Furthermore, BTC contains electronegative oxygen atoms, which withdraw electron density from the benzene ring through resonance effects. This withdrawal of electron density makes the benzene ring less electron‐rich and reduces its ability to donate electrons. Additionally, electron‐withdrawing carboxylic acid groups disrupt the π‐electron system of the benzene ring.^[^ ^62^ ^]^ This disruption reduces the delocalization of π‐electrons within the ring, diminishing its ability to donate electrons. Instead, the carboxylic acid groups exert a strong electron‐withdrawing effect, making BTC more prone to act as an electron acceptor rather than a donor.^[^ ^63^ ^]^ On the other hand, when we specifically delve into the 2Melm‐BTC binary, a significant absorption edge shift is observed compared to all other MOF samples. This indicated the strongest interaction tendency of 2Melm with BTC in the presence of Co^2+^ and Ce^3+^ ions, resulting in better TENG performance. The imidazole rings in 2Melm contain two nitrogen atoms with lone pairs of electrons readily available for donation to other atoms or molecules Figure 2a and Figure S1a (Supporting Information).^[^ ^28^ ^]^ Moreover, the electronic structure of imidazole involves a delocalized π‐electron system. This π‐system consists of a five‐membered ring containing nitrogen atoms, which participate in resonance stabilization, enhancing the electron‐donating capabilities^[^ ^29^, ^30^ ^]^ On the other hand, BDC and BTC contain electron withdrawing carboxylic acid groups that decrease electron density on the benzene rings of these ligands decreasing the availability of transferable charge carriers as shown in Figure 2b,c and Figure S1b,c (Supporting Information).

**Figure 6:**
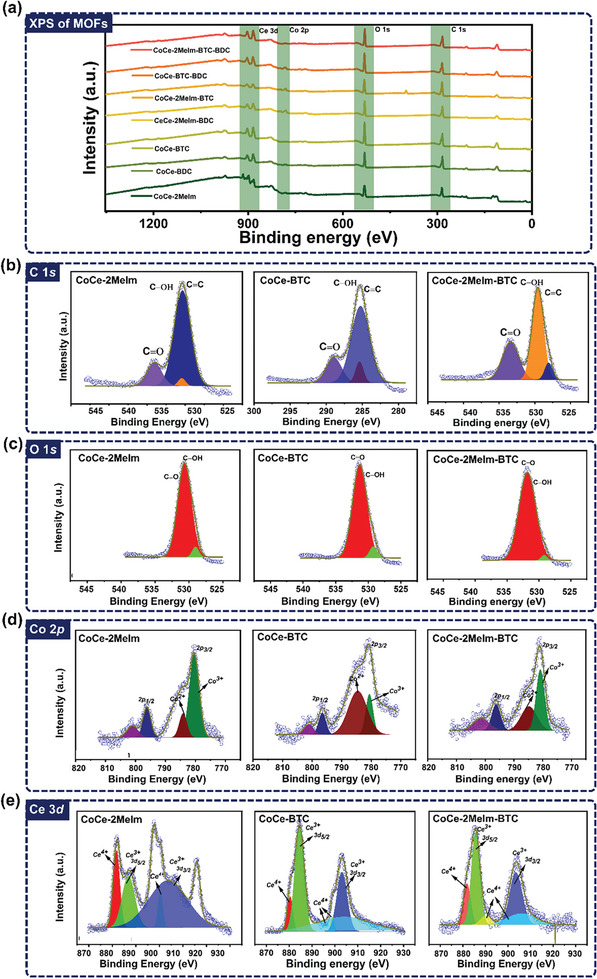
a) XPS survey scan of all MOF samples, b) C 1*s*, c) O 1*s*, d) Co 2*p*, and e) Ce 3*d* of CoCe‐2Melm, CoCe‐BTC, and CoCe‐2Melm‐BTC.

The XPS analysis was conducted to investigate the surface chemical states, composition, and electronic states of elements, as depicted in **Figures** **6** and S2 (Supporting Information). Figure 6a illustrates the survey scan of unary, binary, and ternary ligand‐based CoCe‐MOFs, revealing the presence of C (1s orbital), O (1s orbital), Co (2p orbital), and Ce (3d orbital) within all MOF samples. In the deconvoluted spectrum of C 1s, peaks observed at binding energies of 289.1, 285.3, and 283.9 eV are attributed to the C═O, C─OH, and C═C of organic ligands, respectively.^[^ ^64^, ^65^ ^]^ A shift in the position and intensity of the 285.3 eV peak in binary and ternary ligands suggests potential complex formation,^[^ ^25^ ^]^ as shown in Figure 6b and Figure S2a (Supporting Information). The O 1s spectrum exhibits characteristic peaks at 530.3 and 531.9 eV, associated with ─OH and ═O/M─O (metal‐oxygen) bonds, respectively. The increased intensity of the 531.9 eV peak in binary ligands suggests enhanced metal‐oxygen bond formation due to complex involvement, as demonstrated in Figure 6c and Figure S2b (Supporting Information). A clearer shift is observed in the case of ternary ligands, which might be associated with the formation of more C─OH. Figures 6d and S2c (Supporting Information) depict the deconvoluted Co 2p spectrum of all CoCe‐MOF samples. Two characteristic peaks associated with 2p_3/2_ at 780.5 and 785.5 eV confirm the presence of Co^3+^ and Co^2+^ valence states in the samples. Additionally, a characteristic peak of 2p_1/2_ is observed at 795.5 eV, showcasing a ≈15 eV difference between 2p_3/2_ and 2p_1/2_ orbitals, indicative of Co^3+^ presence.^[^ ^66^ ^]^ Notably, binary and ternary ligand MOFs exhibit some extra peaks in the Co spectrum, potentially linked to Co oxidation and environmental effects, suggesting the formation of CoO_2_, Co_2_O_3,_ as also evident from the XRD data. Similarly, the deconvoluted Ce 3d spectrum in Figures 6e and S2d (Supporting Information) shows bands at 885.3 and 904.2 eV, attributed to Ce^3+^ in Ce 3d_5/2_ and Ce 3d_3/2_ orbitals. Other peaks observed at 881.7, 889.7, and 907.2 eV may relate to Ce^4+^.^[^ ^67^ ^]^ The variation in valence states can induce a tribopolarity effect within materials, leading to surface polarization, ease of electron availability and increased electron donation. Additionally, the variable valence states can affect the surface chemistry leading to the formation of electron‐donating functionalities on the surface. Moreover, various valence states such as Co^3+^/Co^2+^ and Ce^3+^/Ce^+4^ may affect the electron density of the ions which may lead to easier electron donation when the surface comes into contact with an electron‐accepting surface.^[^ ^68^, ^69^ ^]^ Hence, XPS analysis confirms the presence of Co and Ce‐based frameworks within CoCe‐MOFs, showcasing multiple valence states such as Co^3+^/Co^2+^ and Ce^3+^/Ce4+ in unary, binary, and ternary MOFs and their effect on TENG performance.

## 3 TENG Performance Analysis

**Figure 7:**
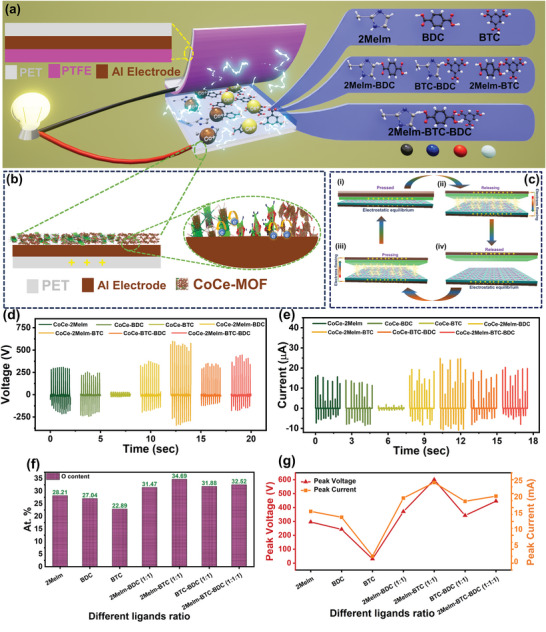
a) Schematic diagram illustrating the CoCe‐MOFs based TENG showing the tribonegative layered structure and combination of various ligands employed, b) tribopositive layered structure in CoCe‐MOF based TENG, c) working mechanism of CoCe‐MOFs based TENG, d) output voltage comparison of CoCe‐MOF samples, e) output current comparison of CoCe‐MOF samples, f) atomic percentage of oxygen atoms in CoCe‐MOFs, and g) comparison of peak voltage and peak current for all MOF samples.

The synthesized CoCe‐MOFs were employed as tribopositive materials in the TENG, while PTFE was used as the tribonegative layer. The complete device fabrication process is explained in the experimental section. **Figure** **7**a illustrates the schematic representation of the device structure and proposed scheme in which different combination of organic ligands are used in bimetallic mofs. Consequently, the Figure 7b shows the tribopositive layered structure in CoCe‐MOF based TENG. Figure 7c shows the electricity generation mechanism during the contact separation. An external horizontal mechanical force was applied to compress the electronegative PTFE layer to elucidate the operational mechanism, causing it to come into contact with the electropositive CoCe‐MOFs layer. In a completely pressed state of the device, charge neutrality occurs due to electrostatic equilibrium between both layers, as shown in Figure 7c(i). During this compression phase, chemical functional groups such as carboxylates, imidazolates, and nitrates within CoCe‐MOFs can initiate the donation of electrons to the PTFE layer. CoCe‐MOFs and PTFE exhibit different electron affinities, and a charge transfer occurs, resulting in the development of oppositely charged surfaces. After removing the external force, the in‐contact layers release each other. This separation instigates the generation of an electric field between them, as shown in Figure 7c(ii). The electric field functions as a driving force, facilitating the transfer of electrons from the PTFE electrode to the CoCe‐MOF electrode. Consequently, this electron transfer leads to the generation of induced charges across both electrodes, effectively counterbalancing the influence of the electric field. These induced charges are subsequently transferred within the external circuit until the external force is eliminated. Once the external force is completely withdrawn, the current within the external circuit diminishes to zero, as illustrated in Figure 7c(iii). In the subsequent phase, under the reapplication of an external force and when both tribolayers are brought back into contact, a reduction in the strength of the electric field occurs in them with a decrease in the separation distance. Accordingly, the induced electrons are compelled to flow in a reverse direction, generating a reverse current within the external circuit, as depicted in Figure 7c(iv).

From this point onward, an electrostatic equilibrium is established when both tribolayers are fully in contact under the influence of the external force. This equilibrium effectively halts the flow of electrons within the external circuit, as delineated in Figure 7c(i). This cyclic process of contact and separation persists, resulting in the generation of alternating signals. Figure 7d,e shows the output voltage and current of seven MOF samples. CoCe‐2Melm‐BTC (598 V, 26.7 µA) demonstrated the higher output performance followed by CoCe‐2Melm‐BTC‐BDC (447 V, 21.7 µA), CoCe‐2Melm‐BDC (377 V, 19.2 µA), CoCe‐BTC‐BDC (356 V, 18.9 µA), CoCe‐2Melm (304 V, 16.8 µA), CoCe‐BDC (250, 13.5 µA), and CoCe‐BTC (31 V, 3.2 µA). Figure 7d,e shows that among the MOFs based on unary ligands, i.e., CoCe‐2MeIm, CoCe‐BDC, and CoCe‐BTC, the MOF synthesized from BTC ligands gives the lowest device performance, whereas the device performances of CoCe‐2MeIm and CoCe‐MOF‐BDC are higher and comparable as is evident from the voltage and current outputs. The device based on ternary ligands, i.e., CoCe‐2‐MeIm‐BDC‐BTC performs better than the MOFs based on unary and binary ligands. In the case of the MOFs derived from binary ligands, i.e., CoCe‐2MeIm‐BDC, CoCe‐2MeIm‐BTC, and CoCe‐BDC‐BTC, the MOFs based on a combination of BTC and 2‐MeIm give very high voltage and current output; actually, this output is the highest among all the devices. It is evident from the device performance that 2‐MeIm and BTC make a perfect combination for making high‐performance MOFs. It is apparent from Figure 7d,e that the MOFs containing 2‐MeIm as a constituent ligand perform better than those lacking this ligand. This might be because of the imidazole ring system's good charge transport properties, which have a delocalized electronic structure.^[^ ^29^ ^]^ The imidazole ring of 2Melm forms coordination bonds with metal ions like cobalt Co^2+^ and cerium Ce^3+^ through its nitrogen atoms. Additionally, the (─COO─) of BTC provides active potential sites for proton (H^+^) transfer, which can influence charge dynamics in the MOFs.

In the case of CoCe‐BTC, the dramatic drop in the output voltage might be associated with the electron‐accepting tendency and bulkier structure of three (─COO─) groups in the BTC ligand.^[^ ^63^ ^]^ This limitation causes poor coordination linkage with metal ions and charge trapping in MOF structure, reducing charge transfer efficiency. In Figure 7f, the oxygen content within all MOF samples is depicted. The highest oxygen content is observed in the 2Melm‐BTC sample, as seen in the XPS survey scan, displaying the strongest peak intensity among the MOF samples. Conversely, BTC demonstrates the weakest oxygen presence. A higher oxygen concentration indicates enhanced electron donation capabilities, positively influencing TENG performance. Figure 7g illustrates comparison graphs for peak voltage and current among all the MOF samples. Additionally, all the MOF samples demonstrated temperature and humidity stabilities as shown in Figures S9 and S10 (Supporting Information), respectively. **Table** **1** provides the comparison of electrical performance of CoCe‐2Melm‐BTC with previous reported reports. Although, many researchers have discussed the effect of incorporating nanoparticles, functionalization, and attaching electron‐withdrawing groups within MOFs but a though investigation regarding the ligand's chemistry was still lacking. This study showcases that identifying the optimal ligand combination is crucial for achieving high‐performance and environmentally stable TENGs. Furthermore, in this study, we have employed a simple, facile solvothermal approach, enhancing the viability of large‐scale production of MOF‐based TENGs.

**Table 1:** Comparison of CoCe‐2Melm‐BTC / PTFE TENG with the reported literatures.

Num.	TENG structure	Device area	Output voltage [V]	Output current [µA]	Power density [µW cm^−2^]	Ref.
1	**ZIF‐7 / KAPTON**	2.5 ×2.5 cm^2^	60	1.1	4.84	[70]
2	**ZIF‐67/PVDF**	2 × 2 cm^2^	119.6	7.7	171	[71]
3	**MIL‐88A / FEP**	2.5 × 2.5 cm^2^	88	2.2	10.12	[72]
4	**MIL‐125(0.25%MOF/Ecoflex)**	2 × 3 cm^2^	305	13	150	[73]
5	**ZIF‐62 / Teflon**	2.5 × 2.5 cm^2^	62	1.4	6.05	[11]
6	**Alpha Cyclodextrin MOF/Teflon**	2 × 2 cm^2^	152	1.2	8	[74]
7	**ZIF‐8**	2.5 × 2.5 cm^2^	164	7	245	[52]
8	**Al foil/PDMS/Silk fibroin/NF‐MOF**	2 × 2 cm^2^	215	10	263	[75]
9	**CoCe‐2Melm‐BTC / PTFE**	**2.5 × 2 cm^2^**	**598**	**26.7**	**603.72**	**Our work**

**Figure 8:**
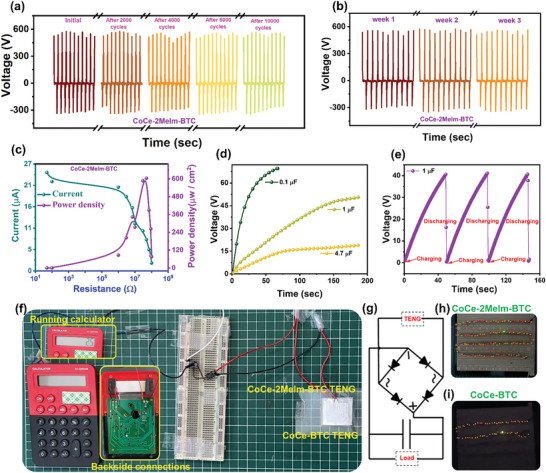
a) cycling stability test of CoCe‐2Melm‐BTC for 10 000 consecutive cycles, b) long‐term stability of CoCe‐2Melm‐BTC for 3 weeks, c) the instantaneous power density graph of CoCe‐2Melm‐BTC, d) charging curves of various commercial capacitors using CoCe‐2Melm‐BTC in TENG, e) continuous charging and discharging curve for 1 µF using CoCe‐2Melm‐BTC in TENG, f) for practical performance comparison, the applicability of CoCe‐2Melm‐BTC and CoCe‐BTC based TENGs to run the calculator along with circuit connections, g) circuit diagram to power the electronic devices using TENG, h) LEDs glowing using CoCe‐2Melm‐BTC TENG and, i) LEDs glowing using CoCe‐BTC TENG.

Afterward, the stability of the device was investigated by running the device for 10 000 consecutive cycles at an applied frequency of 5 Hz, as shown in **Figure** **8**a. The device demonstrated excellent stability against the applied mechanical strokes. Furthermore, TENG's stability performance was investigated weekly by checking the output voltages after one, two, and three weeks, as shown in Figure 8b. The TENG device demonstrated very stable behavior after three weeks. While considering the TENG devices for practical applications, the power density is an important parameter to assess their efficiency and applicability. At an applied frequency of 5 Hz, the power density of the CoCe‐2Melm‐BTC based TENG was calculated by employing various load resistances ranging from 50 Ω to 100 MΩ and the corresponding drop in current values was recorded as shown in Figure 8c. The TENG device showed a peak power density of 603.72 µW cm^−2^ at 5 MΩ load resistance. Figure 8d demonstrates the charging of commercial capacitors with capacities of 0.1, 1, and 4.7 µF using the CoCe‐2Melm‐BTC. The TENG charged the 0.1 µF capacitor up to 69.2 V in 68 s, the 1 µF capacitor up to 51.3 V in 186 s, and the 4.7 µF capacitor up to 18.8 V in 186 s. Subsequently, Figure 8e presents the charging/discharging curve of the 1 µF capacitor. Under the applied frequency of 5 Hz, the device effectively charged the capacitor, while the discharging curve in this scenario was obtained by attaching a small stopwatch to the charging capacitor. Afterward, for the practical demonstration, CoCe‐2Melm‐BTC and CoCe‐BTC based TENGs were utilized to run the calculator as illustrated in Figure 8f. Figure 8f also shows the circuit connections, where a full‐wave bridge rectifier and a 0.22 µF capacitor were integrated in the circuit. Notably, the CoCe‐2Melm‐BTC based TENG powered a clearly displayed calculator screen. In contrast, the CoCe‐BTC based TENG, due to its lowest outperformance, resulted in a faded screen. Video S1 (Supporting Information) demonstrates the comparison of two devices to run the calculator. Figure 8g shows a general circuit diagram followed to power the electronic devices. Furthermore, we employed these TENG devices for illuminating LEDs, as depicted in Figure 8h,i. The CoCe‐2Melm_BTC‐based TENG successfully lit up ≈86 LEDs, whereas the utilization of the CoCe‐BTC TENG device resulted in the illumination of only 32 LEDs. Video S2 (Supporting Information) illustrates the comparative LED illumination demonstrations for the two TENG devices.

## 4 Conclusion

In this study, we conducted a ligand‐based investigation on MOFs employing 2Melm, BDC, and BTC ligands in unary, binary, and ternary combinations. We aimed to determine the most effective ligand combination for TENG devices. Consequently, the CoCe‐2Melm‐BTC combination displayed the most promising results, indicating a synergistic effect between the imidazole ring and‐ (─COO─) groups. This is associated with the lone pairs on nitrogen atoms, a fully active delocalized π‐electron system, and the nitrogen's electronegativity in imidazole rings. Specifically, CoCe‐2Melm‐BTC demonstrated superior performance, recording results of 598 V and 26.7 µA, while the CoCe‐BTC exhibited the lowest performance with 31 V and 3.2 µA. The lower performance of CoCe‐BTC is attributed to the bulkiness of electron‐withdrawing (─COO─) groups and their effect on the benzene ring's π‐electron system. Furthermore, we found that MOFs based on binary and ternary ligands performed better than those based on unary ligands. This thorough analysis clarifies the vital role of MOF chemistry as well as the impact of ligands within TENGs. The study suggests that optimizing the triboelectric charge for improved TENG performance can be greatly assisted by an understanding of the mixed ligand chemistry in MOFs.

## 5 Experimental Section

### Materials

The precursor materials for the synthesis of CoCe‐MOFs were acquired from Sigma Aldrich, including 2‐methylimedazole (2Melm), terephthalic acid (1,4‐denzene‐dicarboxylic acid (BDC), trimesic acid (1,−3,−5,‐benzene‐tricarboxylic acid (BTC), cobalt (II) nitrate hexahydrate (Co(NO_3_)_2_.6H_2_O), and cerium (III) nitrate hexahydrate (Ce(NO_3_)_3_.6H_2_O). The supplier of polyethylene terephthalate (PET) sheets was Film Bank in South Korea, and aluminum tapes were purchased from ELEPARTS Electronics.

### Synthesis of CoCe‐MOFs

A facile solvothermal procedure was used to synthesize the CoCe‐MOFs with some modfifications.^[^ ^35^, ^36^ ^]^ Initially, 0.25 mmol of metallic salt precursors, such as Co (NO_3_)_2_.6H_2_O (0.54 g) and Ce (NO_3_)_3_.6H_2_O (0.54 g) were dissolved separately in 10 ml of DMF. To get the maximum homogeneity, magnetic stirring followed by probe sonication for 20 min was carried out at room temperature. Afterward, 1.0 mmol of organic ligands with various combinations such as 2Melm (1.05 g), BDC (1.05 g), BTC (1.05 g), 2Melm: BDC (0.525 g: 0.525 g), BDC: BTC (0.525 g: 0.525 g), 2Melm: BTC (0.525 g: 0.525 g) and 2Melm: BDC: BTC (0.35 g: 0.35 g: 0.35 g) were dissolved separately in 40 ml DMF following the same procedure as described previously. To maintain the consistent synthesis conditions and uniform distribution of interacting species, the reaction mixtures were subjected to magnetic stirring for 30 min, followed by probe‐sonication for 15 min. Afterward, the resultant solutions were poured into a Teflon‐lined hydrothermal autoclave reactor. For the chemical reaction, the autoclave reactor was kept in a muffle furnace at 130 °C temperature for 18 h, followed by self‐cooling. Before pouring in the precursor solutions, the autoclave was thoroughly washed with HCl, ethanol, and DI water. Once the reaction had finished, precipitates of varying colors were obtained for varied ligand combinations. The obtained precipitates were washed several times with ethanol and DI‐water separated through centrifugation (4000 RMP for 7 min each) and dried at 70 °C overnight in a hot air oven. The CoCe‐MOF samples with different organic ligands were named as CoCe‐2Melm, CoCe‐BDC, CoCe‐BTC, CoCe‐2Melm‐BDC, CoCe‐2Melm‐BTC, CoCe‐BTC‐BDC, and CoCe‐2Melm‐BDC‐BTC respectively, as shown in Figure 1.

### Assembly of CoCe‐MOF‐based TENG Device

In the fabrication process of the TENG device, the electropositive layer was composed of CoCe‐MOF with different organic ligands, while polytetrafluoroethylene (PTFE) was employed as an electronegative layer. The top electrode, having dimensions of 2.5 cm × 2 cm, was formed by sticking a PTFE sheet on an aluminum substrate. Conversely, the bottom electrode, with identical dimensions, was made by depositing CoCe‐MOF with different organic ligands on an aluminum substrate. A PET sheet was utilized as the housing material throughout the fabrication process. The PET sheet was meticulously adhered to opposing facets of the aluminum electrodes using double‐sided tape. This flexible PET sheet performed a dual role, acting as an electrical insulator and a structural support for the top and bottom electrodes. It offered indispensable assistance during the phases of contact and separation when subjected to external mechanical excitation forces. For electrical connectivity, conductive wires were directed from the top and bottom electrodes and then linked to an oscilloscope through a 100 MΩ probe. A programmable linear motor was employed to impart external mechanical excitation forces of different frequencies such as 2, 4, and 5 Hz.^[^ ^76^ ^]^

### Instrumentation

The examination of the crystalline nature of CoCe‐MOFs was carried out by an X‐ray diffractometer (Panalytical X'PERT PRO model) equipped with a Cu Kα target having a wavelength (λ) of 1.5046 Å and diffraction angle (2θ) ranged from 10° to 80°. The surface morphology and elemental compositions were assessed using a scanning electron microscope with a built‐in Energy Dispersive X‐ray Spectrometer (EDX) testing facility (MIRA‐3 TESCON FESEM). High‐resolution transmission electron microscopy (HR‐TEM) images and EDX mapping were captured utilizing the TEM (Talos F200X G2) housed at Jeju National University. To prepare the sample for HR‐TEM analysis, a minor quantity of the MOF powders was dispersed in ethanol, followed by 15 min of bath sonication. Subsequently, the resulting dispersions were drop‐casted onto a 3 mm carbon film‐coated Cu grid with a mesh size of 200 (SPI). XPS spectra were measured at 1.0 × 10^−9^ Torr base pressure with a monochromatic Al‐Kα X‐ray source (ESCALAB 250XI, Thermo Fisher Scientific). Ultraviolet‐visible (UV–vis) absorption spectra were measured in DMF on an Agilent single‐beam UV–vis absorption spectrometer. FT‐IR spectra analyses were conducted using a Fourier Transform infrared spectrometer (ALPHA II), spanning a scan range of 400–4000 cm⁻¹. A programmable linear motor (NTI AG LinMot) was employed to apply the mechanical excitation force. The measurements of electrical output voltage and stability performance of CoCe‐MOFs based TENG were conducted employing a precision instrumentation oscilloscope (Agilent Technologies DSO6052A), whereas the probe impedance was maintained at 100 MΩ. Conversely, the electrical current signals and instantaneous power values were meticulously recorded through a source/measurement unit (KEYSIGHT B2902A). Notably, the determination of power values for the TENG devices was achieved through a mathematical expression *P  =  I^2^R*, where *I* represents the electric current, while *R* denotes the external load resistance.

## Conflict of Interest

The authors declare no conflict of interest.

## Supporting information

Supporting Information

Supplemental Video 1

Supplemental Video 2

## Data Availability

The data that support the findings of this study are available from the corresponding author upon reasonable request.

## References

[1] T. Li , M. Wu , J. Xu , R. Du , T. Yan , P. Wang , Z. Bai , R. Wang , S. Wang , Nat. Commun. 2022, 13, 6771. 36351950 10.1038/s41467-022-34385-4 PMC9646691

[2] E. Lattanzi , E. Regini , A. Acquaviva , A. Bogliolo , Comput. Commun. 2007, 30, 2976.

[3] S. Kim , J. Shen , M. Ahad , Int. J. Appl. Sci. Technol. 2015, 5, 1

[4] D. Ma , G. Lan , M. Hassan , W. Hu , S. K. Das , IEEE Commun. Surv. Tutorials 2020, 22, 1222.

[5] S. Nand , 2020.

[6] F. F. Ahmad , C. Ghenai , M. Bettayeb , Sustainable Energy Technol. Assessments 2021, 47, 101430.

[7] Y. Gai , Y. Jiang , Z. Li , Nano Energy 2023, 116, 108787.

[8] A. K. Aliyana , G. Stylios , Adv. Sci. 2023, 10, 2304232. 10.1002/advs.202304232 PMC10582424 37607119

[9] M. Shanbedi , H. Ardebili , A. Karim , Prog. Polym. Sci. 2023, 144, 101723.

[10] Y. M. Wang , X. Zhang , C. Liu , L. Wu , J. Zhang , T. Lei , Y. Wang , X. B. Yin , R. Yang , Nano Energy 2023, 107, 108149.

[11] G. Khandelwal , N. P. Maria Joseph Raj , S. J. Kim , J. Mater. Chem. A 2020, 8, 17817.

[12] C. Liu , Q. Sun , L. Lin , J. Wang , C. Zhang , C. Xia , T. Bao , J. Wan , R. Huang , J. Zou , C. Yu , Nat. Commun. 2020, 11, 4971. 33009408 10.1038/s41467-020-18776-z PMC7532534

[13] L. Chai , J. Pan , Y. Hu , J. Qian , M. Hong , L. Chai , Y. Hu , J. Qian , J. Pan , M. Hong , Small 2021, 17, 2100607. 10.1002/smll.202100607 34245231

[14] K. Ikigaki , K. Okada , Y. Tokudome , T. Toyao , P. Falcaro , C. J. Doonan , M. Takahashi , Angew. Chem. 2019, 131, 6960. 10.1002/anie.201901707 30924218

[15] J. Xiong , W. Wang , H. Du , Z. Zhou , A. Zhao , L. Mi , S. Chen , RSC Adv. 2022, 12, 30051. 36329932 10.1039/d2ra05537f PMC9583627

[16] R. A. Shaukat , Q. M. Saqib , J. Kim , H. Song , M. U. Khan , M. Y. Chougale , J. Bae , M. J. Choi , Nano Energy 2022, 96, 107128.

[17] Y. M. Wang , X. Zhang , C. Liu , L. Wu , J. Zhang , T. Lei , Y. Wang , X. B. Yin , R. Yang , Nano Energy 2023, 107, 108149.

[18] L. Chen , H. F. Wang , C. Li , Q. Xu , Chem. Sci. 2020, 11, 5369. 34094065 10.1039/d0sc01432j PMC8159423

[19] T. Wang , Q. Zhu , Q. Zhu , Q. Yang , S. Wang , L. Luo , Nanoscale Adv. 2022, 4, 4314. 36321143 10.1039/d2na00379a PMC9552755

[20] M. Toyabur Rahman , M. Sazzadur Rahman , H. Kumar , K. Kim , S. Kim , M. T. Rahman , M. S. Rahman , H. Kumar , K. Kim , S. Kim , Adv. Funct. Mater. 2023, 33, 2303471.

[21] S. M. S Rana , M. A. Zahed , M. R. Islam , O. Faruk , H. S. Song , S. H. Jeong , J. Yeong Park , Chem. Eng. J. 2023, 473, 144989.

[22] T. Mazur , B. A. Grzybowski , Chem. Sci. 2017, 8, 2025. 28451320 10.1039/c6sc02672a PMC5398273

[23] J. A. Villajos , M. Bienert , N. Gugin , F. Emmerling , M. Maiwald , Mater. Adv. 2023, 4, 4226.

[24] X. Z. Xie , Y. R. Zhang , Y. J. Wu , X. B. Yin , Y. Xia , J. Phys. Chem. C 2023, 127, 1220.

[25] G. Sahoo , H. S. Jeong , S. M. Jeong , ACS Appl. Mater. Interfaces 2023, 15, 21097. 37075253 10.1021/acsami.3c01580

[26] A. Babu , L. Bochu , S. Potu , R. Kaja , N. Madathil , M. Velpula , A. Kulandaivel , U. K. Khanapuram , R. K. Rajaboina , H. Divi , P. Kodali , B. Ketharachapalli , R. Ammanabrolu , ACS Sustainable Chem. Eng. 2023, 11, 16806.

[27] G. Khandelwal , N. Prashanth , M. J. Raj , S.‐J. Kim , G. Khandelwal , N. P. Maria , J. Raj , S.‐J. Kim , Adv. Energy Mater. 2021, 11, 2101170.

[28] B. Hachuła , M. Nowak , J. Kusz , J. Chem. Crystallogr. 2010, 40, 201.

[29] G. Durán‐Solares , W. Fugarolas‐Gómez , N. Ortíz‐Pastrana , H. López‐Sandoval , T. O. Villaseñor‐Granados , A. Flores‐Parra , P. J. Altmann , N. Barba‐Behrens , J. Coord. Chem. 2018, 71, 1935.

[30] C. I. H. Ashby , C. P. Cheng , T. L. Brown , J. Am. Chem. Soc. 1978, 100, 6057.

[31] W. Yang , X. Wang , H. Li , J. Wu , Y. Hu , Z. Li , H. Liu , Nano Energy 2019, 57, 41.

[32] K. Wu , D. Wang , Q. Fu , T. Xu , Q. Xiong , S. Gouse , Inorg. Chem. 2024, 63, 11135. 38829208 10.1021/acs.inorgchem.4c00787

[33] J. Hao , Z. Jiang , H. Abbas Khan , O. El Tall , A. Farooq , Fuel 2022, 318, 123638.

[34] R. Aniruddha , V. M. Shama , I. Sreedhar , C. M. Patel , J. Clean. Prod. 2022, 350, 131478.

[35] Y. Li , W. Han , R. Wang , L. T. Weng , A. Serrano‐Lotina , M. A. Bañares , Q. Wang , K. L. Yeung , Appl. Catal. B Environ. 2020, 275, 119121.

[36] Y. Zheng , Y. Zheng , Q. Zhao , Q. Zhao , C. Shan , C. Shan , S. Lu , S. Lu , Y. Su , Y. Su , R. Han , R. Han , C. Song , N. Ji , N. Ji , D. Ma , D. Ma , Q. Liu , Q. Liu , ACS Appl. Mater. Interfaces 2020, 12, 28139. 32423199 10.1021/acsami.0c04904

[37] Q. Zhang , X. Yang , J. Yao , J. Cheng , J. Chem. 2021, 2021, 32131960.

[38] M. Wang , F. Li , J. Dong , X. Lin , X. Liu , D. Wang , W. Cai , J. Environ. Chem. Eng. 2022, 10, 107892.

[39] M. Wang , F. Li , W. Cai , J. Energy Inst. 2022, 105, 157.

[40] J. Li , Y. Kang , Z. Lei , P. Liu , Appl. Catal. B Environ. 2023, 321, 122029.

[41] T. Xie , L. Wang , H. Wang , C. Cao , C. Tang , X. Hu , Sep. Purif. Technol. 2024, 330, 125231.

[42] X. Zha , R. Xi , Y. Wu , J. Xu , Y. Yang , Sensors 2022, 22, 6891. 36146240 10.3390/s22186891 PMC9501041

[43] X. Yang , S. H. Yuan , A. Zou , H. Drake , Y. I. Zhang , J. U. Qin , A. Alsalme , H.‐C. Zhou , X. Yang , S. Yuan , L. Zou , H. Drake , Y. Zhang , J. Qin , H.‐C. Zhou , A. Alsalme , Angew. Chem., Int. Ed. 2018, 57, 3927. 10.1002/anie.201710019 29451952

[44] S. Choi , T. Kim , H. Ji , H. J. Lee , M. Oh , J. Am. Chem. Soc. 2016, 138, 14434. 27728969 10.1021/jacs.6b08821

[45] J. Narciso , E. V. Ramos‐Fernandez , J. J. Delgado‐Marín , C. W. Affolter , U. Olsbye , E. A. Redekop , Microporous Mesoporous Mater. 2021, 324, 111310.

[46] L. Ai , Y. Luo , W. Huang , Y. Tian , J. Jiang , Int. J. Hydrogen Energy 2022, 47, 12893.

[47] G. Zeng , Y. Chen , L. Chen , P. Xiong , M. Wei , Electrochim. Acta 2016, 222, 773.

[48] S. R. Patil , M. Y. Chougale , J. Kim , R. A. Shaukat , M. Noman , Q. M. Saqib , M. U. Khan , T. D. Dongale , J. Bae , Energy Technol. 2022, 10, 2200876.

[49] A. A. Meshram , S. M. Sontakke , Mater. Today Proc. 2021, 46, 6201.

[50] M. Belaye , A. M. Taddesse , E. Teju , M. Sanchez‐Sanchez , J. M. Yassin , ACS Omega 2023, 8, 23860. 37426255 10.1021/acsomega.3c02290 PMC10324055

[51] Z. Kejie , Z. Chencheng , F. Yangjie , T. Meng , M. Huixiu , J. Yun , W. Qi , Z. Kejie , Z. Chencheng , F. Yangjie , T. Meng , M. Huixiu , J. Yun , W. Qi , Acta Mater. Compos. Sin. 2022, 39, 3891.

[52] G. Khandelwal , A. Chandrasekhar , N. Prashanth Maria Joseph Raj , S.‐J. Kim , G. Khandelwal , A. Chandrasekhar , N. P. Maria Joseph Raj , S. Kim , Adv. Energy Mater. 2019, 9, 1803581.

[53] J. Sun , X. Yu , S. Zhao , H. Chen , K. Tao , L. Han , Inorg. Chem. 2020, 59, 11385. 32799472 10.1021/acs.inorgchem.0c01157

[54] W.‐W. Zhong , F. D. Firuzabadi , Y. Hanifehpour , X. Zeng , Y.‐J. Feng , K.‐G. Liu , S. W. Joo , A. Morsali , P. Retailleau , Inorganics 2023, 11, 184.

[55] B. Zhang , J. Zhang , C. Liu , X. Sang , L. Peng , X. Ma , T. Wu , B. Han , G. Yang , RSC Adv. 2015, 5, 37691.

[56] H. F. Greer , Y. Liu , A. Greenaway , P. A. Wright , W. Zhou , Cryst. Growth Des. 2016, 16, 2104.

[57] J. G. Santaclara , F. Kapteijn , J. Gascon , M. A. Van Der Veen , CrystEngComm 2017, 19, 4118. 10.1039/c7fd00029d 28678276

[58] E. A. Dolgopolova , A. J. Brandt , O. A. Ejegbavwo , A. S. Duke , T. D. Maddumapatabandi , R. P. Galhenage , B. W. Larson , O. G. Reid , S. C. Ammal , A. Heyden , M. Chandrashekhar , V. Stavila , D. A. Chen , N. B. Shustova , J. Am. Chem. Soc. 2017, 139, 5201. 28316244 10.1021/jacs.7b01125

[59] B. Bhattacharya , D. K. Maity , A. Layek , S. Jahiruddin , A. Halder , A. Dey , S. Ghosh , C. Chowdhury , A. Datta , P. P. Ray , D. Ghoshal , CrystEngComm 2016, 18, 5754.

[60] Y. Zhang , J. Guo , L. Shi , Y. Zhu , K. Hou , Y. Zheng , Z. Tang , Sci. Adv. 2017, 3, 1701192. 10.1126/sciadv.1701162 PMC5562422 28835929

[61] J. Xu , J. Liu , Z. Li , X. Wang , Y. Xu , S. Chen , Z. Wang , New J. Chem. 2019, 43, 4092.

[62] D. Lei , J. Xue , X. Peng , S. Li , Q. Bi , C. Tang , L. Zang , Appl. Catal. B Environ. 2021, 282, 119578.

[63] D. Yuan , X. Chen , Z. Li , C. Fang , J. Ding , H. Wan , G. Guan , Appl. Surf. Sci. 2021, 569, 151089.

[64] M. H. Kim , S. J. Park , T. J. Ha , Energy Environ. Mater. 2023, e12675.

[65] G. Sahoo , S. R. Polaki , S. Ghosh , N. G. Krishna , M. Kamruddin , J. Power Sources 2018, 401, 37.

[66] Y. Kang , Y. H. Zhang , Q. Shi , H. Shi , D. Xue , F. N. Shi , J. Colloid Interface Sci. 2021, 585, 705. 33121757 10.1016/j.jcis.2020.10.050

[67] Y. Liao , Y. Xiao , Z. Li , X. Zhou , J. Liu , F. Guo , J. Li , Y. Li , Small 2023, 20, 2307685. 10.1002/smll.202307685 37946630

[68] M. T. Greiner , L. Chai , M. G. Helander , W. M. Tang , Z. H. Lu , Adv. Funct. Mater. 2012, 22, 4557.

[69] R. Smoluchowski , Phys. Rev. 1941, 60, 661.

[70] G. Khandelwal , N. P. Maria Joseph Raj , S. J. Kim , Adv. Funct. Mater. 2020, 30, 1910162.

[71] M. H. Memon , U. E. S. Amjad , A. Mir , M. Mustafa , ACS Appl. Electron. Mater. 2024, 6, 2178.

[72] G. Khandelwal , N. P. Maria Joseph Raj , V. Vivekananthan , S. J. Kim , iScience 2021, 24, 102064. 33554068 10.1016/j.isci.2021.102064 PMC7859291

[73] A. Kakim , A. Nurkesh , B. Sarsembayev , D. Dauletiya , A. Balapan , Z. Bakenov , A. Yeshmukhametov , G. Kalimuldina , A. Kakim , A. Nurkesh , B. Sarsembayev , D. Dauletiya , A. Balapan , Z. Bakenov , A. Yeshmukhametov , G. Kalimuldina , Adv. Sens. Res. 2024, 2300163.

[74] S. Hajra , M. Sahu , A. M. Padhan , I. S. Lee , D. K. Yi , P. Alagarsamy , S. S. Nanda , H. J. Kim , Adv. Funct. Mater. 2021, 31, 2101829.

[75] Z. Chen , Y. Cao , W. Yang , L. An , H. Fan , Y. Guo , J. Mater. Chem. A 2022, 10, 799.

[76] R. A. Shaukat , S. Ameen , Q. M. Saqib , M. Y. Chougale , J. Kim , S. R. Patil , M. Noman , H. K. Kim , J. Bae , J. Mater. Chem. A 2023, 11, 14800.

