# Supplementary material for: Controlling Triboelectric Charge of MOFs by Leveraging Ligands Chemistry
Source: Adv Sci (Weinh). 2024 Jul 12;11(35):2404993. doi: 10.1002/advs.202404993 (PMC11425226; doi:10.1002/advs.202404993)
Supplement: Supplementary file 1 — Supporting Information [file ADVS-11-2404993-s003.docx]

**Supporting Information**

Controlling Triboelectric Charge of MOFs by Leveraging Ligands Chemistry

Muhammad Noman^†^, Qazi Muhammad Saqib^†^, Shahid Ameen, Swapnil R. Patil, Chandrashekhar S. Patil, Jungmin Kim, Youngbin Ko, BongSoo Kim and Jinho Bae*

^†^These authors contributed equally to this work.


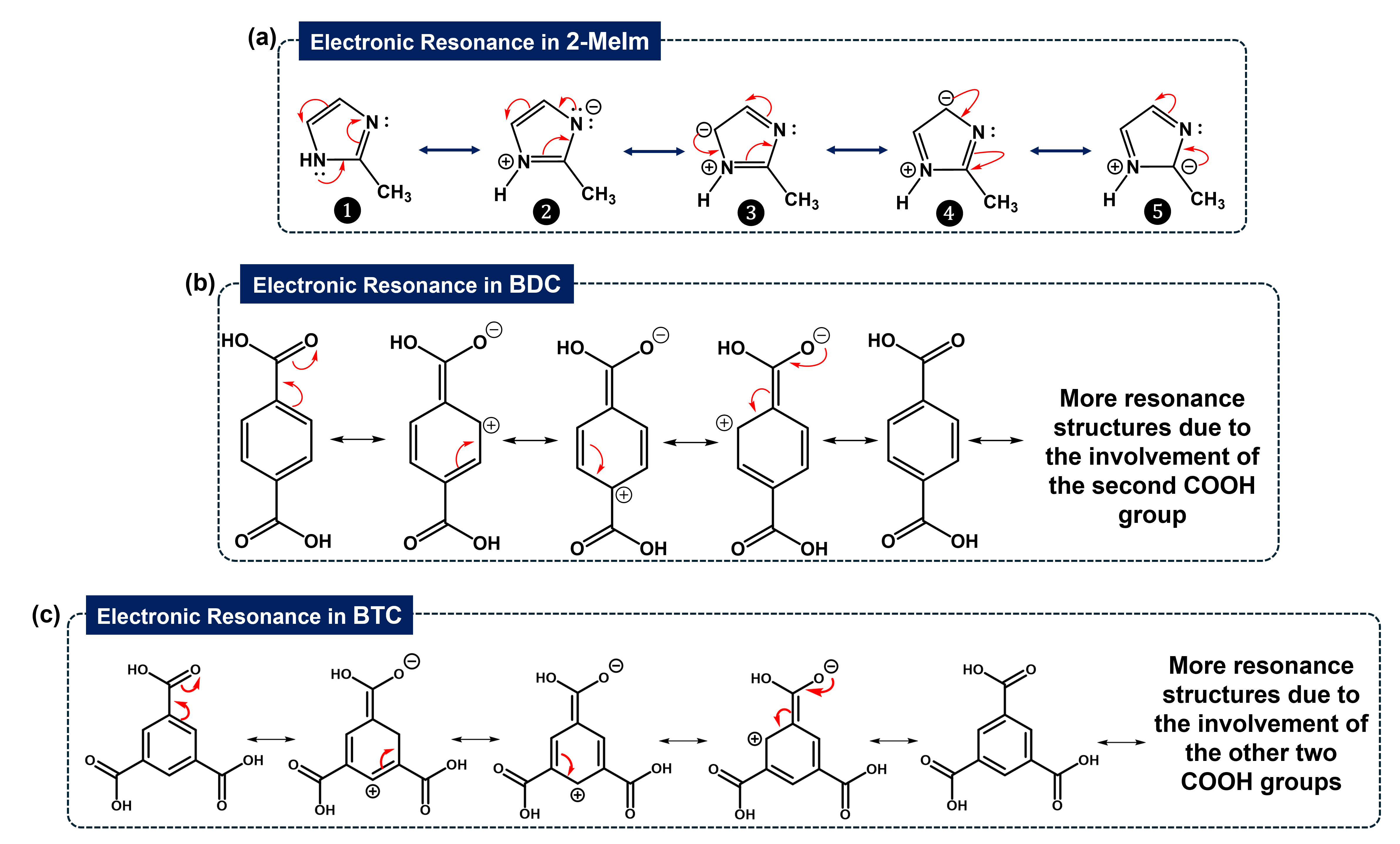


**Figure S1**. Electronic resonance in 2-MeIm, BDC and BTC ligands


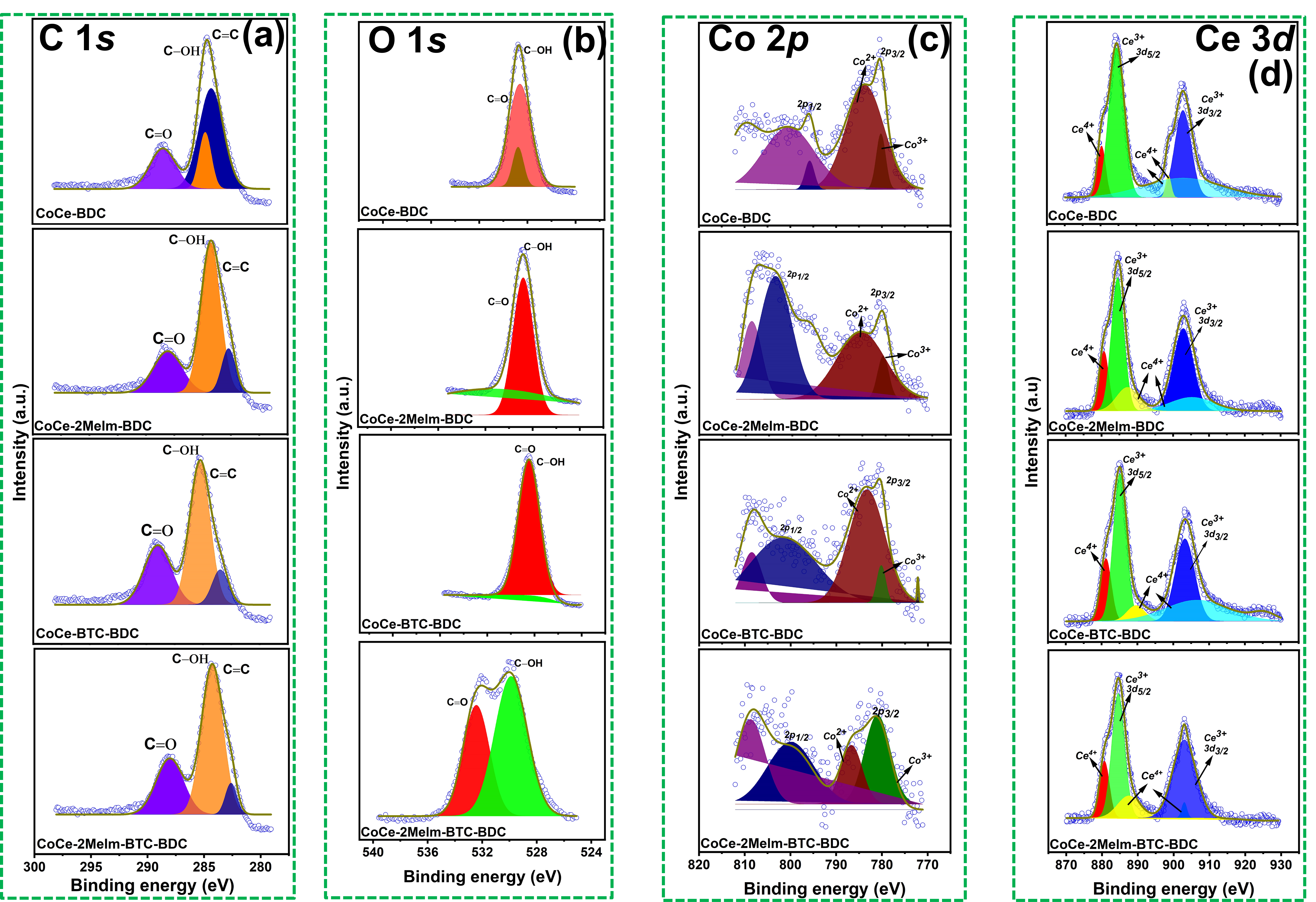


**Figure S2.** Deconvoluted spectra of CoCe-BDC, CoCe-2Melm-BDC, CoCe-BTC-BDC and CoCe-2Melm-BTC-BDC; (a) C 1s (b) O 1s, (c) Co 2p, and (d) Ce 3d.


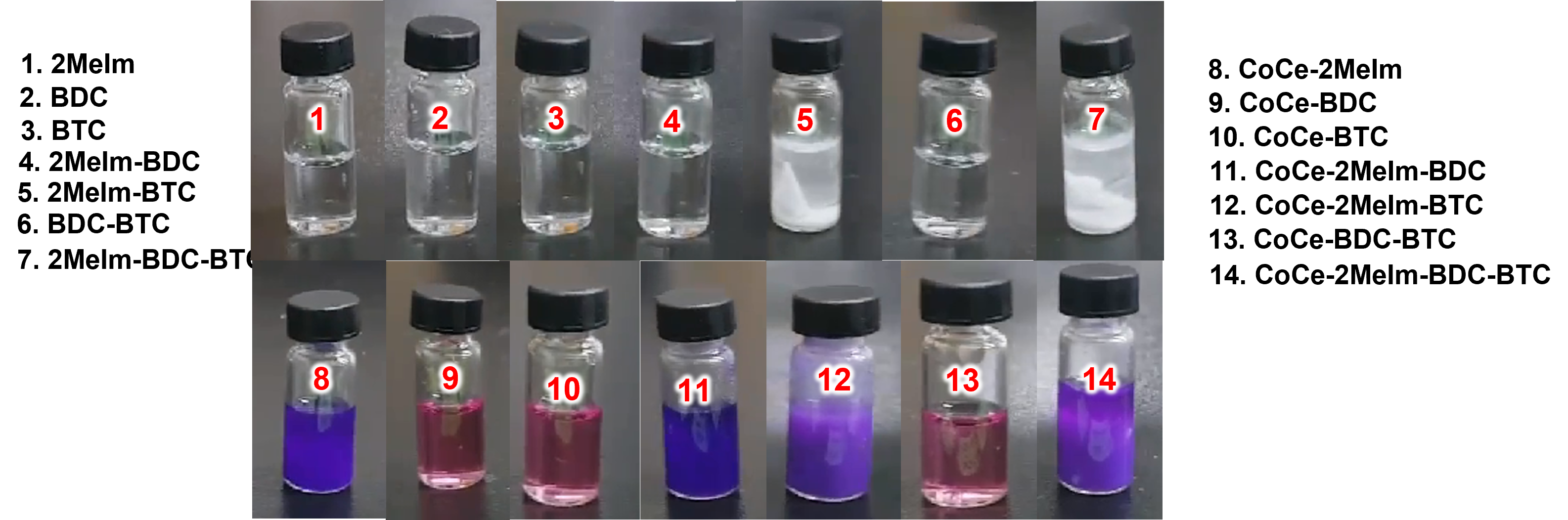


**Figure S3.** Solutions exhibiting different colors due to the various combinations of ligands and ionic salts for UV-vis spectroscopy analysis.


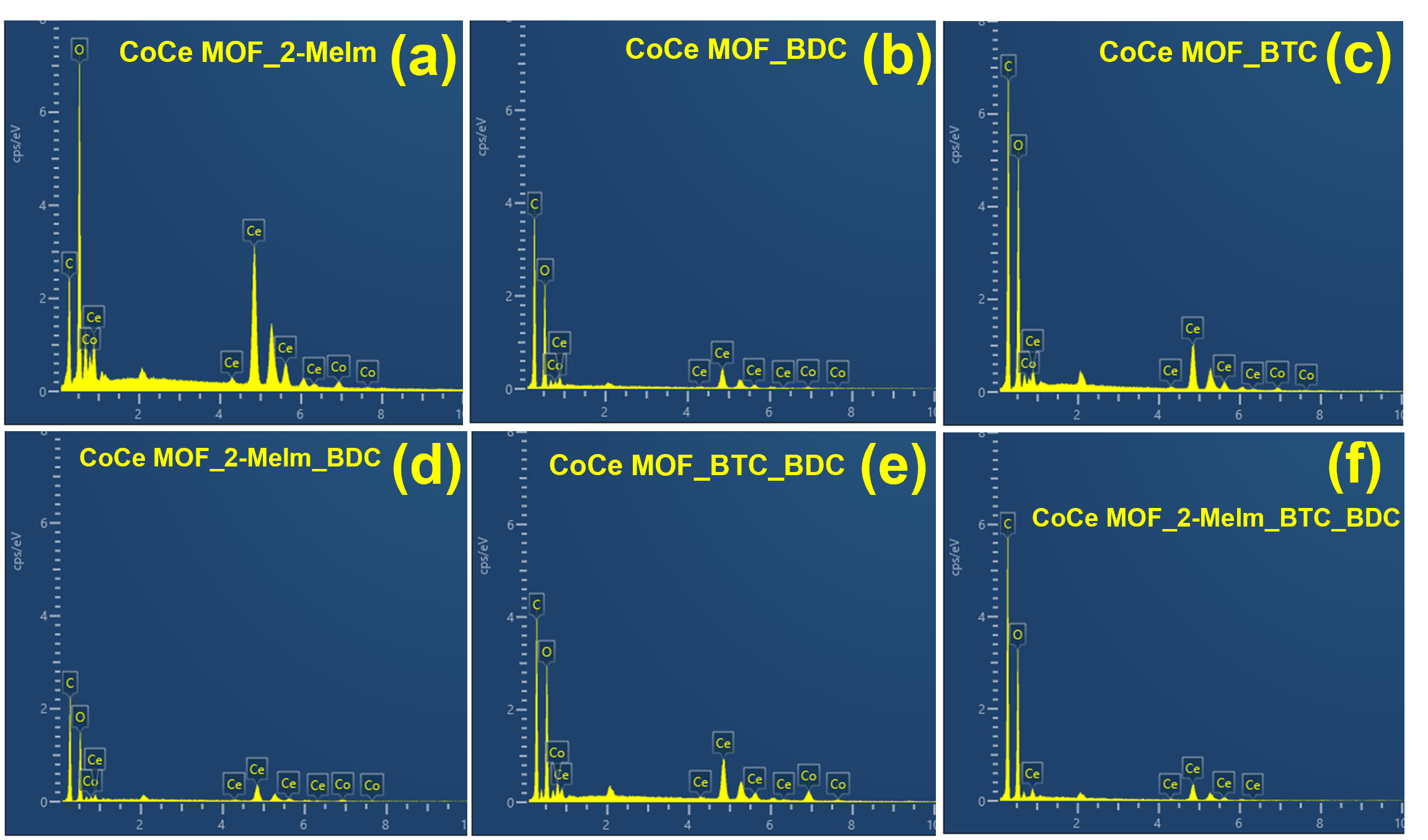


**Figure S4.** EDX mapping spectrums of all MOF samples.


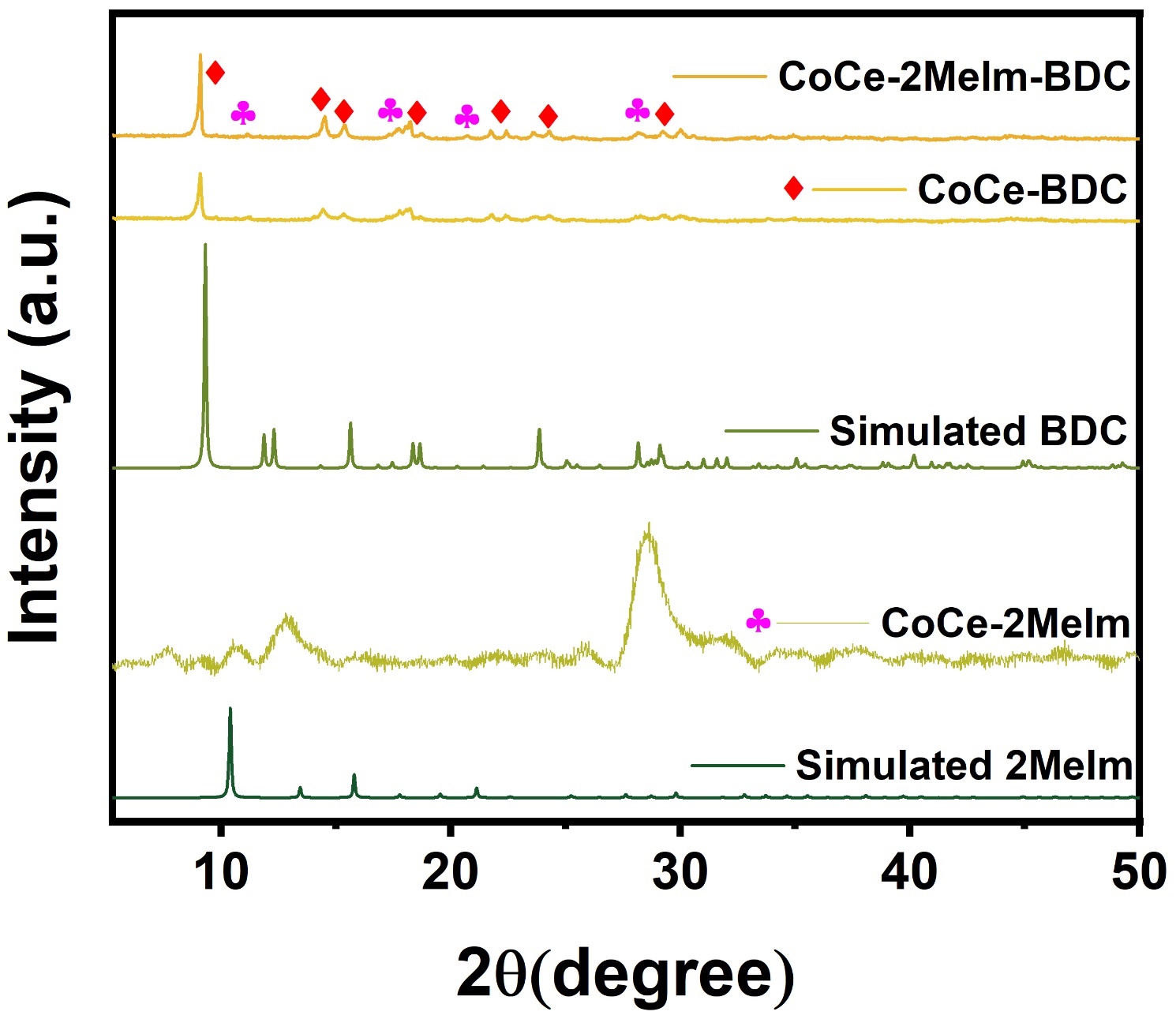


**Figure S5.** XRD patterns of simulated 2Melm, CoCe-2Melm, simulated BDC, CoCe-BDC and CoCe-2Melm-BDC MOF-on-MOF structure.


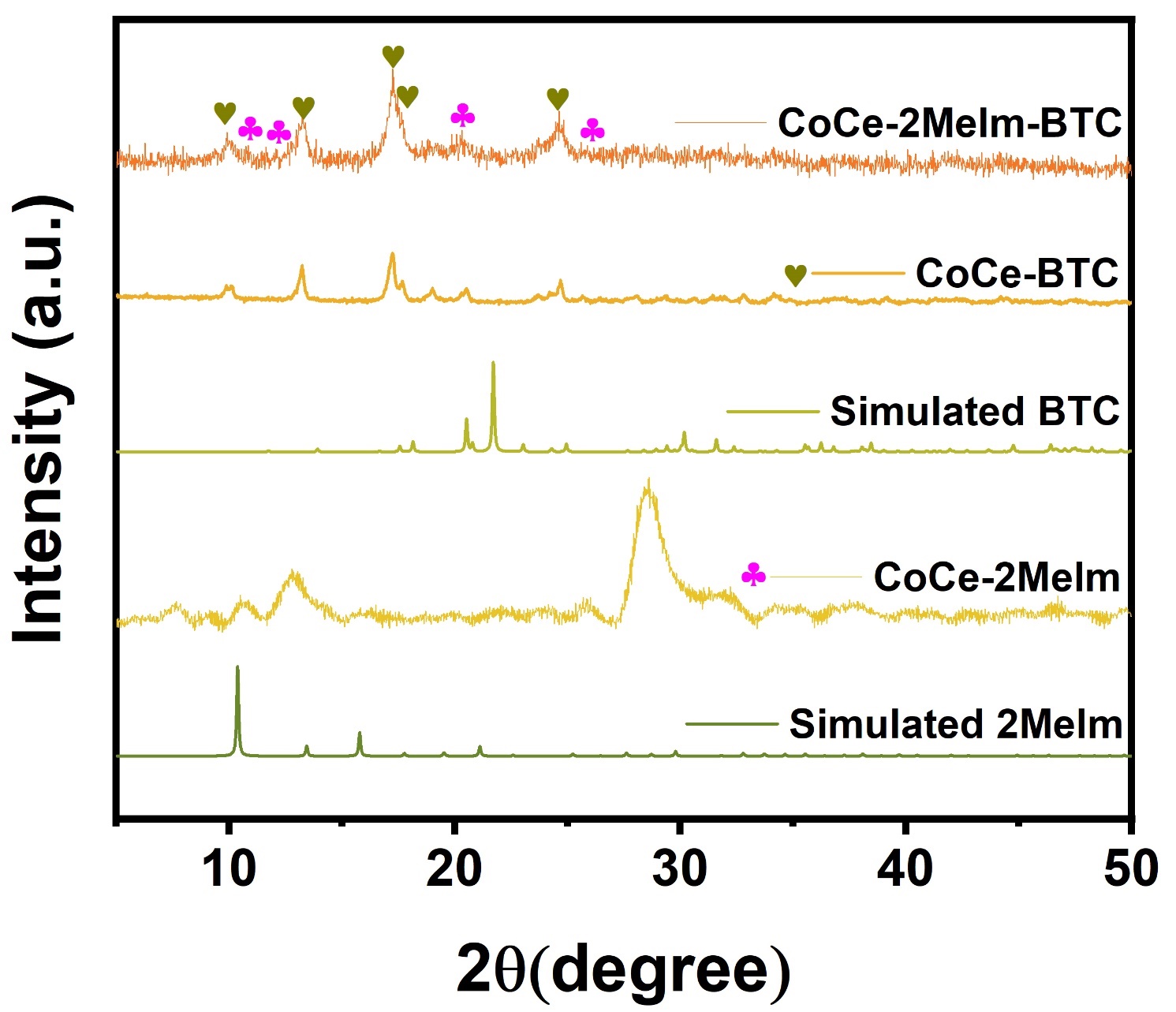


**Figure S6.** XRD patterns of simulated 2Melm, CoCe-2Melm, simulated BTC, CoCe-BTC and CoCe-2Melm-BTC MOF-on-MOF structure.


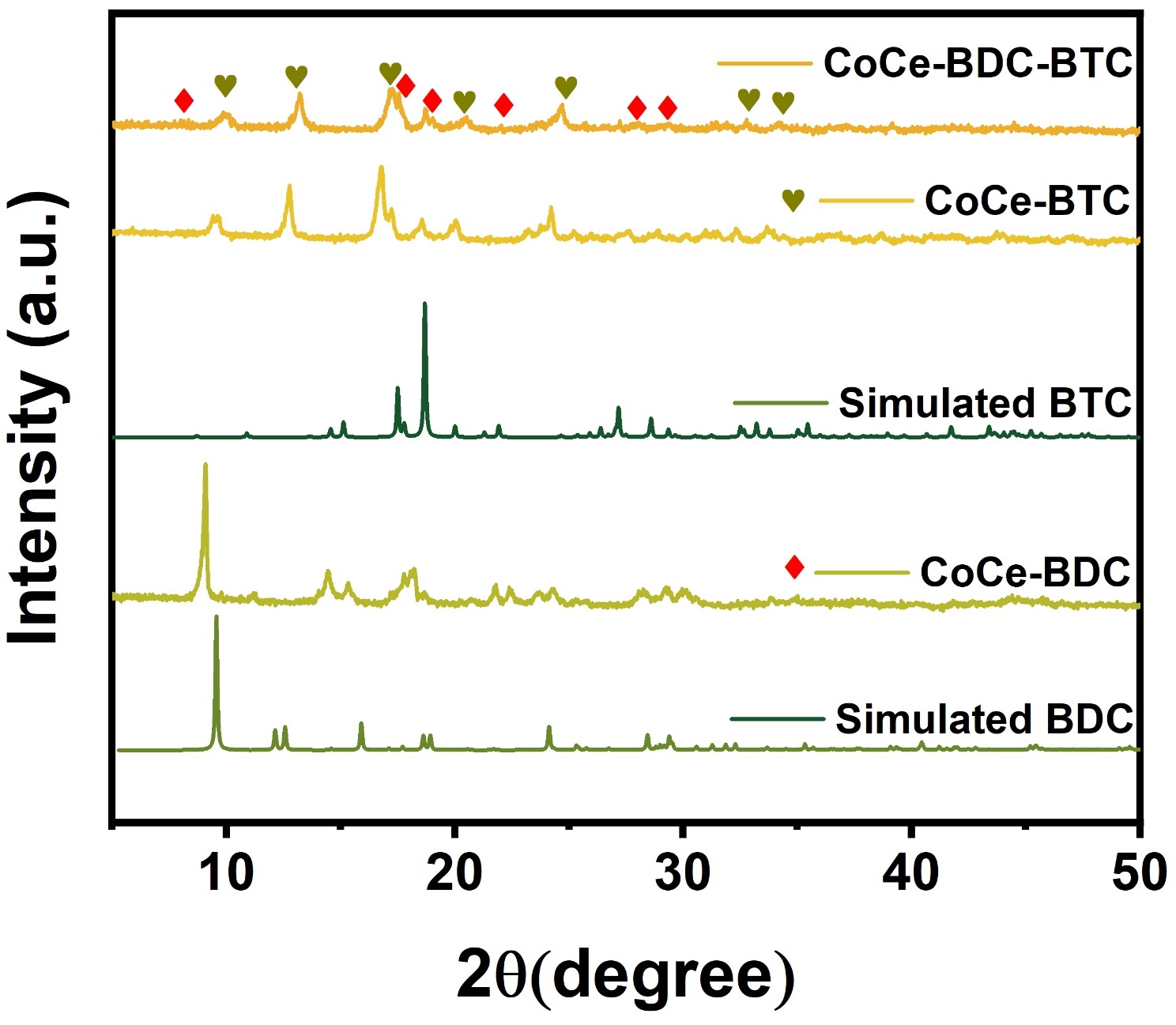


**Figure S7.** XRD patterns of simulated BDC, CoCe-BDC, simulated BTC, CoCe-BTC and CoCe-BDC-BTC MOF-on-MOF structure.


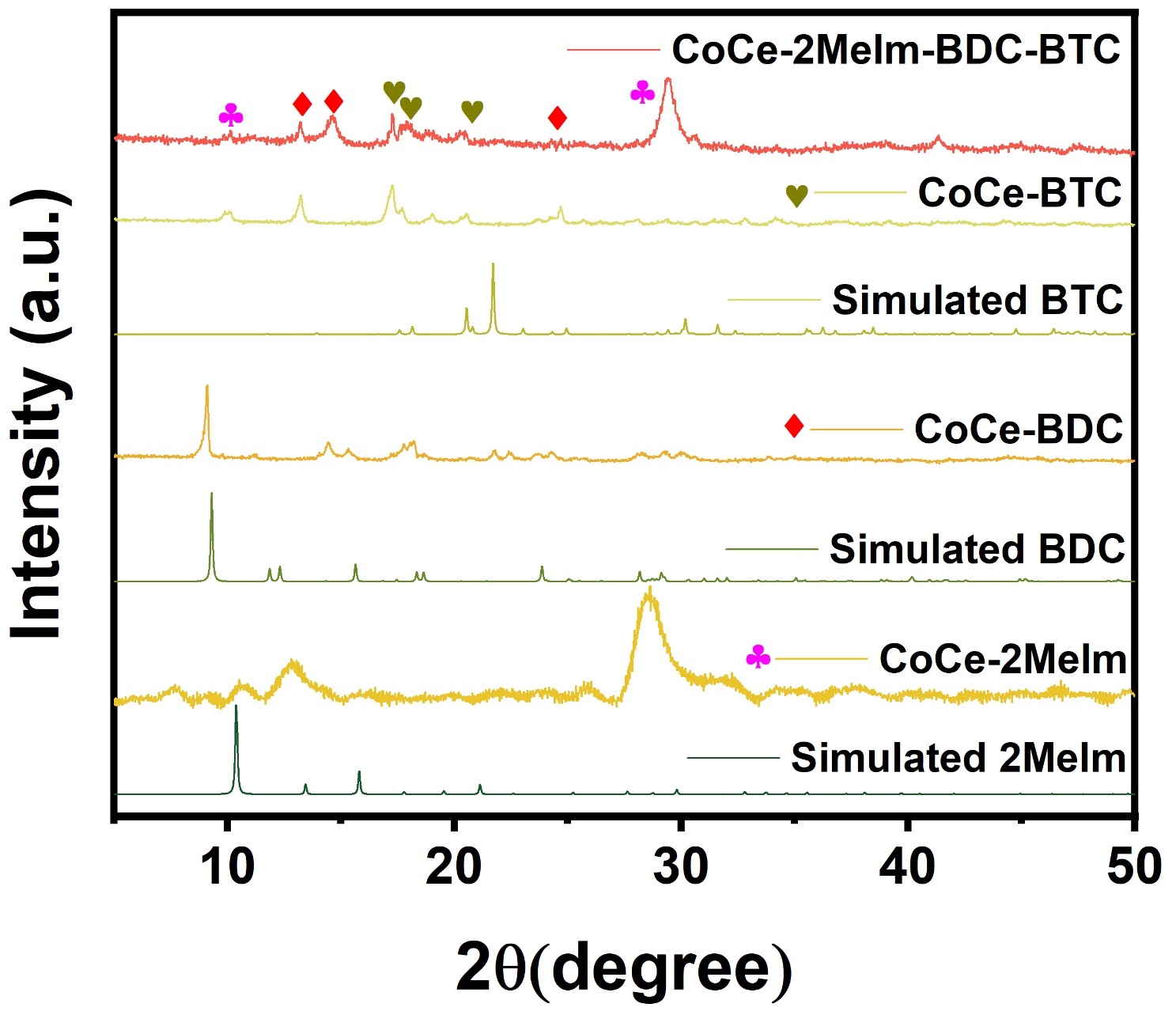


**Figure S8.** XRD patterns of simulated 2Melm, CoCe-2Melm, simulated BDC, CoCe-BDC, simulated BTC, CoCe-BTC and CoCe-2Melm-BDC-BTC MOF-on-MOF structure.


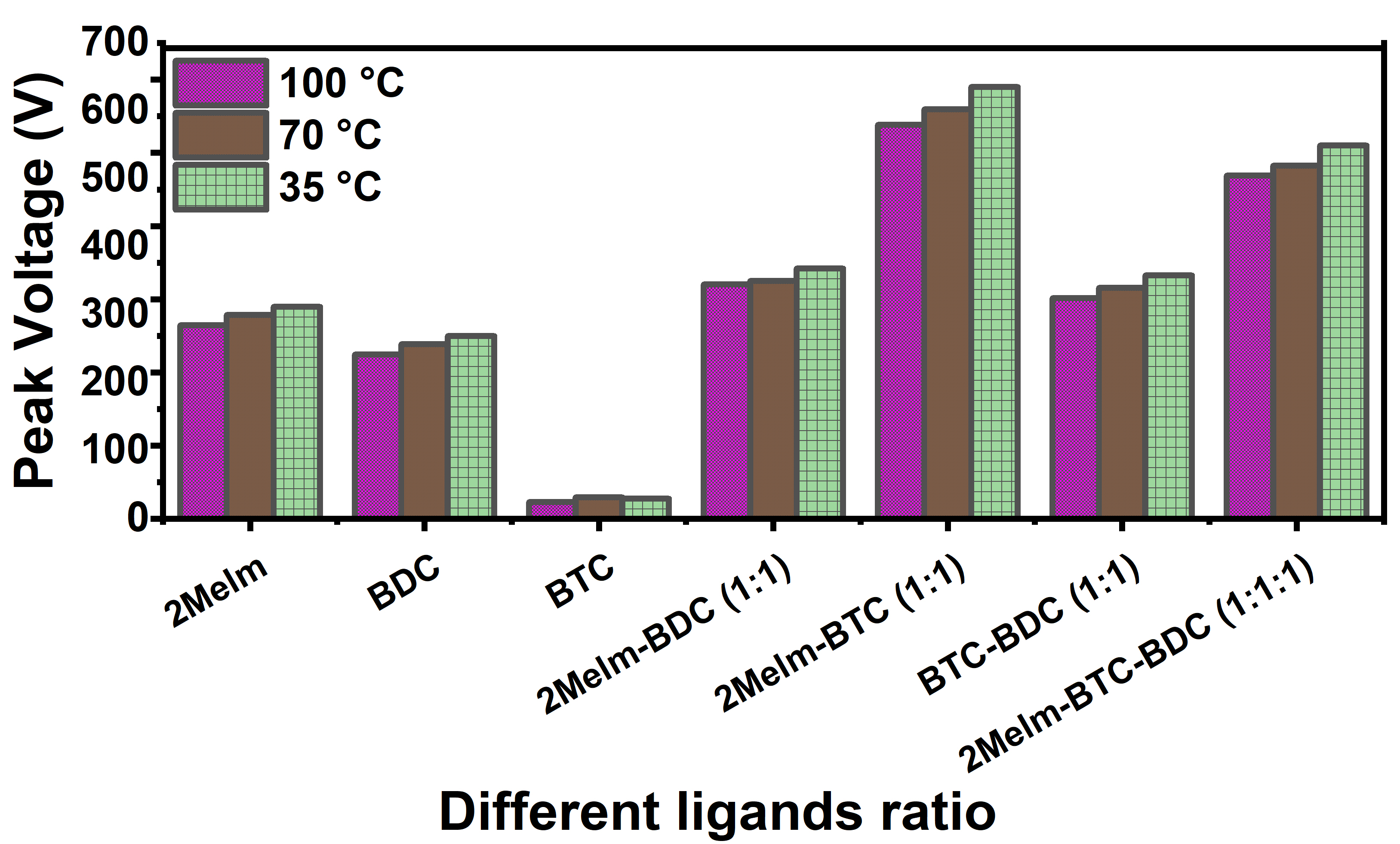


**Figure S9.** Peak output voltage of all MOF samples by hand slapping at various temperatures.


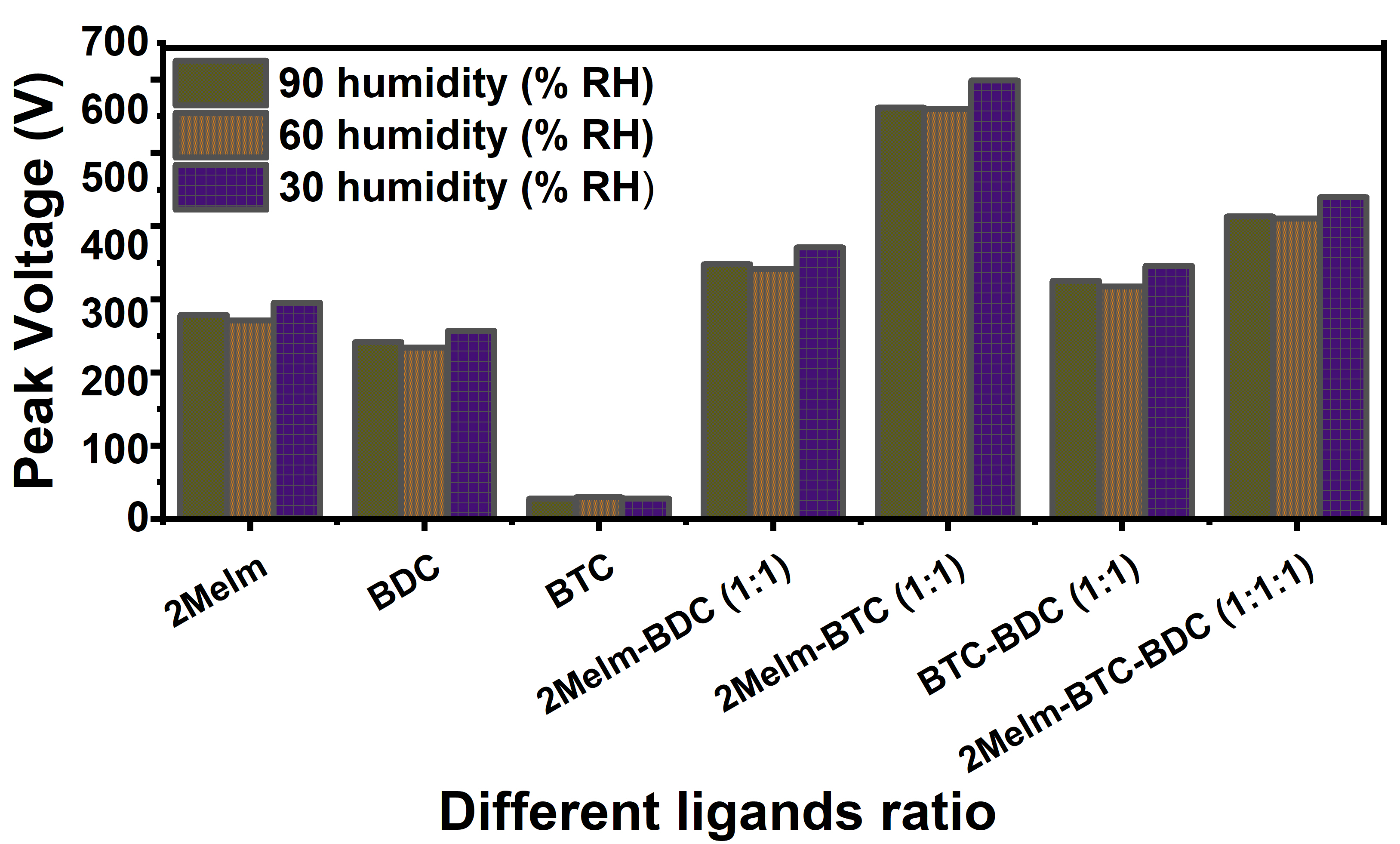


**Figure S10.** Peak output voltage of all MOF samples at various relative humidity levels.
